# Evaluating the global, regional, and national impact of syphilis: results from the global burden of disease study 2019

**DOI:** 10.1038/s41598-023-38294-4

**Published:** 2023-07-14

**Authors:** Tao Chen, Bo Wan, Mingfang Wang, Su Lin, Yinlian Wu, Jiaofeng Huang

**Affiliations:** 1 The First Affiliated Hospital, Fujian Medical University, Fuzhou, Fujian China; 2 Faculty of Life Sciences and Medicine, King’s College London, London, UK; 3 Department of Hepatology, Hepatology Research Institute, The First Affiliated Hospital, Fujian Medical University, No. 20, Chazhong Road, Taijiang District, Fuzhou, 350005 Fujian China; 4 Fujian Clinical Research Center for Liver and Intestinal Diseases, The First Affiliated Hospital, Fujian Medical University, Fuzhou, Fujian China; 5 Department of Hepatology, National Regional Medical Center, Binhai Campus of the First Affiliated Hospital, Fujian Medical University, Fuzhou, Fujian China

**Keywords:** Infectious diseases, HIV infections

## Abstract

Syphilis is a global public health concern. This study aimed to assess the global and regional burden of syphilis from 1990 to 2019. Disease burden was evaluated using disability-adjusted life-years (DALYs) and prevalence. Data were extracted from the 2019 global burden of disease Study, an open database available for download. Age-standardized rates (ASR) and estimated annual percentage changes (EAPC) were calculated to evaluate the syphilis burden over time. In 2019, the total number of prevalent cases of syphilis was 49.71 million worldwide. The ASR of prevalence was stable from 1990 to 2019 with an EAPC of 0.00 (95% CI − 0.10–0.11). The number of DALYs caused by syphilis was 7.36 million in 2019, reflecting a reduction of 16.38% compared with that in 1990 (8.80 million). The ASR of DALYs exhibited a decreasing trend from 1990 to 2019 (EAPC =  − 1.01; 95% CI − 1.19 to − 0.84), with the highest rates observed in the younger age group (< 14 years old). In 2019, the highest ASR of DALYs was found in low sociodemographic index (SDI) regions (239.21/100,000), and the lowest in high SDI regions (3.14/100,000). Generally, the ASR of DALYs decreased as the SDI increased. The top three countries with the highest ASR of DALYs for syphilis were the Solomon Islands, Equatorial Guinea, and Liberia. While the global prevalence of syphilis remained persistently high from 1990 to 2019, there has been a recent decrease in the ASR of DALYs. Increased attention should be dedicated to younger populations and regions characterized by low SDIs.

## Introduction

Syphilis is a sexually transmitted infection (STIs) caused by *Treponema pallidum* that has imposed a significant global burden in the past few decades^1^. Syphilis not only affects the genitourinary system^2^ but also impairs other systems, including the neurological, cardiovascular, ocular, or liver systems^3,4^. Patients diagnosed with syphilis may experience discrimination, stigma, exclusion, and pressure from society and family^5^. During the 69th World Health Assembly in 2016, a target was set to reduce the global incidence of syphilis by 90% from 2018 to 2030^6^. Nevertheless, there has been a recent indication of an increase in the global prevalence of syphilis. To achieve the target set by the 69th World Health Assembly, countries need to revise their policies regarding syphilis and especially focus on high-risk populations.

The burden and rate of syphilis show variations based on time, location, and population. There has been a discernable rise in the prevalence of syphilis in Europe and Australia^7,8^. Additionally, syphilis has extended its reach beyond urban areas and is spreading to rural or remote areas^8^. A report from South–East Asia revealed the emergence of a subsequent wave of syphilis in the past 15 years^9^. Most studies have focused on examining the burden of syphilis within specific regions and did not extend their investigation to encompass global changes^7–9^. A recent systematic review revealed that the frequency of maternal and congenital syphilis in indigenous people varies but tends to be higher compared to that in their non-indigenous counterparts. Data from China indicated a substantial increase in syphilis diagnoses over time, particularly among less developed cities and older women^10^. These groups had received comparatively less attention. In a study conducted in the United States, a similar finding was reported, revealing an increase in the burden of syphilis among older adults; notably, this population has always been overlooked or neglected^11^.

Previous studies have indicated that the prevention of mother-to-child transmission (MTCT) could be key to reducing the burden of syphilis. In 2008, an estimated 1.36 million pregnant women were reported to have active syphilis, resulting in serious complications, including pelvic inflammatory disease, premature birth, low birth weight, stillbirth, and neonatal infection^12^. Antenatal screening and treatment of syphilis have played a crucial role in safeguarding the well-being of both the mother and newborn, as well as promoting child health^13^. However, the association between syphilis, economic status, and age group is not well established. Identifying the groups most affected by the disease and formulating effective programs and policies that specifically target these populations are essential for syphilis control.

The global burden of disease study (GBD) is an open database providing a systematic scientific assessment of the prevalence and disability-adjusted life-years (DALYs) of 369 diseases worldwide^14^. Based on this database, studies have been conducted to assess the burden of HIV and acute viral hepatitis. These studies have consistently demonstrated that HIV and acute viral hepatitis remain significant contributors to the loss of healthy life^15,16^. A systematic analysis of the burden of syphilis is still lacking. In this study, we used the 2019 GBD data to analyze the temporal trends and burden of syphilis. The results of our study will enhance our knowledge of the burden of syphilis and will assist in the formulation of strategies for effective prevention and control of the disease.

## Methods

### Study data

Syphilis data from 1990 to 2019 were obtained from the Global Health Data Exchange query tool (http://ghdx.healthdata.org/gbd-results-tool). In the GBD 2019 study, all statistics are presented as values with 95% uncertainty intervals (UI) after 100,000 simulations. The following selection criteria were applied to select the data for analysis. First, the study period was set to 1990–2019. Second, the location name was set to include “Global”, “the 21 geographic locations”, “sociodemographic index (SDI) areas”, and “the 204 countries or territories”^17^. Third, the cause was set to “syphilis”. Fourth, “prevalence” and “DALYs” were chosen as the evaluation index of syphilis burden. Finally, age was segregated into four categories: up to 14, 15–49, 50–69, and 70 + years.

### Definitions

#### Age-standardized rate (ASR)^18^

Age-standardized rate (ASR) was based on the GBD 2019 global age (standard population). The ASR in this study mainly included the ASR of prevalence and ASR of DALYs.

#### Disability-adjusted life-years (DALYs)

The global burden of syphilis was estimated in terms of disability-adjusted life years (DALYs) in the GBD 2019 study, which was defined as the sum of years of life lost and years lived with a disability^18^.

#### Sociodemographic index (SDI)

The sociodemographic index (SDI) is a summary indicator of economic and societal development and was constructed using education, average income, and overall fertility rate under the age of 25^19^. In the GBD 2019, countries and territories were grouped into five SDI quintiles: low, low-middle, middle, high-middle, and high.

#### Estimated annual percentage change (EAPC)

The estimated annual percentage change (EAPC) and 95% confidence intervals (CI) were calculated as the average annual percentage change in ASR from 1990 to 2019. If the EAPC and lower 95% CI limit were both positive, the ASR was considered to have an upward trend. Conversely, if the EAPC and the upper 95% CI limit were both negative, the ASR was considered to have a decreasing trend. Otherwise, ASR was regarded to be consistent over time.

### Joinpoint regression analysis

Joinpoint regression analyses were performed using Joinpoint software version 5.0, which was obtained from the US National Cancer Institute (https://surveillance.cancer.gov/joinpoint/). This software was used to calculate the annual percent change (APC) and average annual percentage change (AAPC). The Joinpoint regression technique has the advantage of characterizing the trend of disease burden changes at many stages.

### Statistical analysis

Data were downloaded from the GBD database and statistical analyses were performed using R software version 4.2.2 (https://www.R-project.org/). Subgroup analysis was performed according to sex, age, SDI, and the 21 geographic locations.

## Results

### Worldwide burden estimates of syphilis

**Figure 1:**
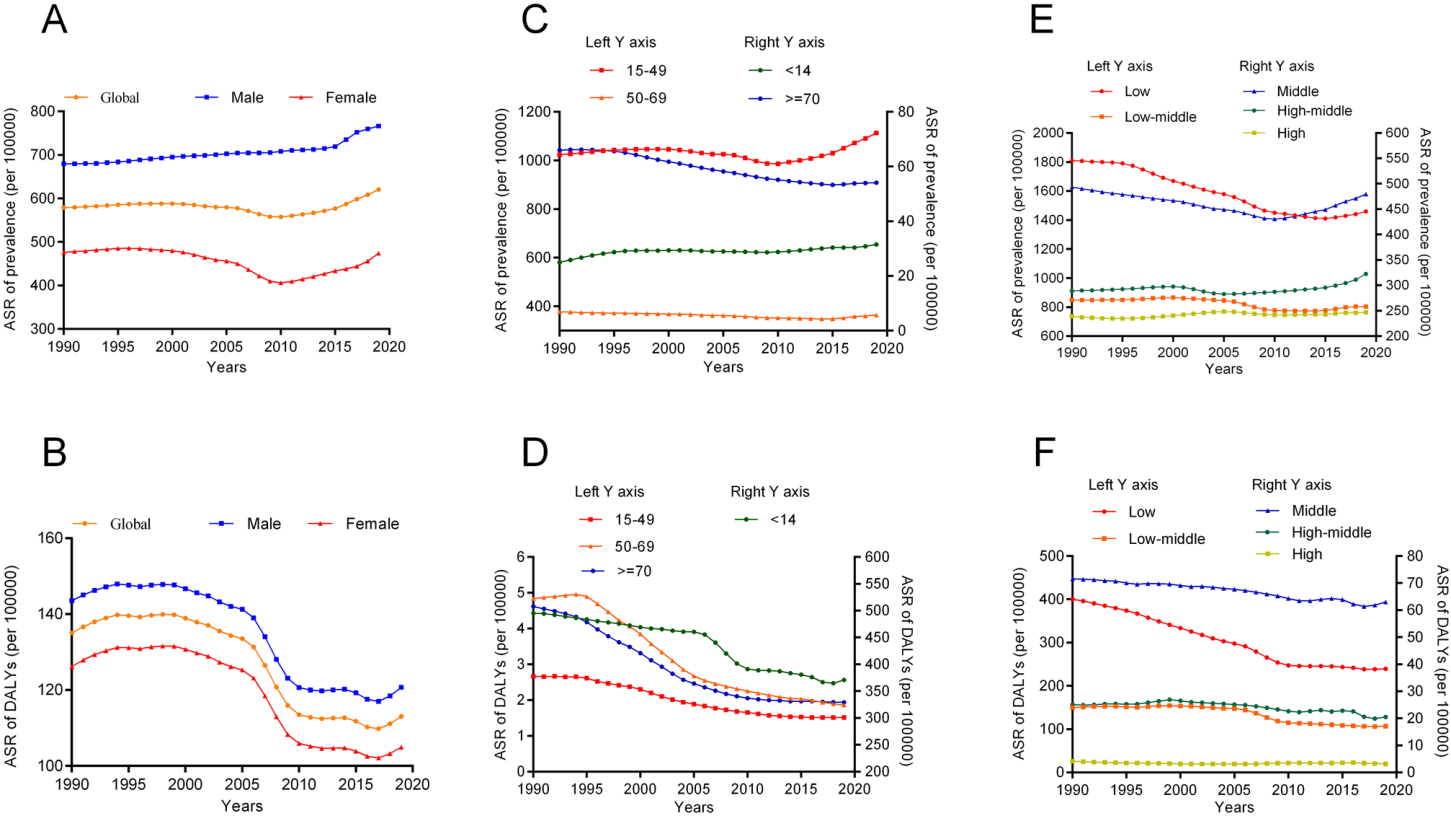
Trend of ASR of syphilis from 1990 to 2019. (**A**) ASR of prevalence grouped by sex; (**B**) ASR of DALYs grouped by sex; (**C**) ASR of prevalence grouped by age, age 15–49 and 50–69 groups are depicted on the left axis, while other age groups are depicted on the right Y-axis; (**D**) ASR of DALYs grouped by age, age 15–49, 50–69, and ≥ 70 groups are depicted on the left axis, while age group < 14 years is depicted on the right Y-axis; (**E**) ASR of prevalence grouped by SDI levels, and low and low middle SDI groups are depicted on the left axis, while other SDI groups are depicted on the right Y-axis. (**F**) ASR of DALYs grouped by SDI levels, and low and low middle SDI groups are depicted on the left axis, while other SDI groups are depicted on the right Y-axis. *DALYs* disability-adjusted life years, *ASR* age-standardized rate, *SDI* sociodemographic index.

**Figure 2:**
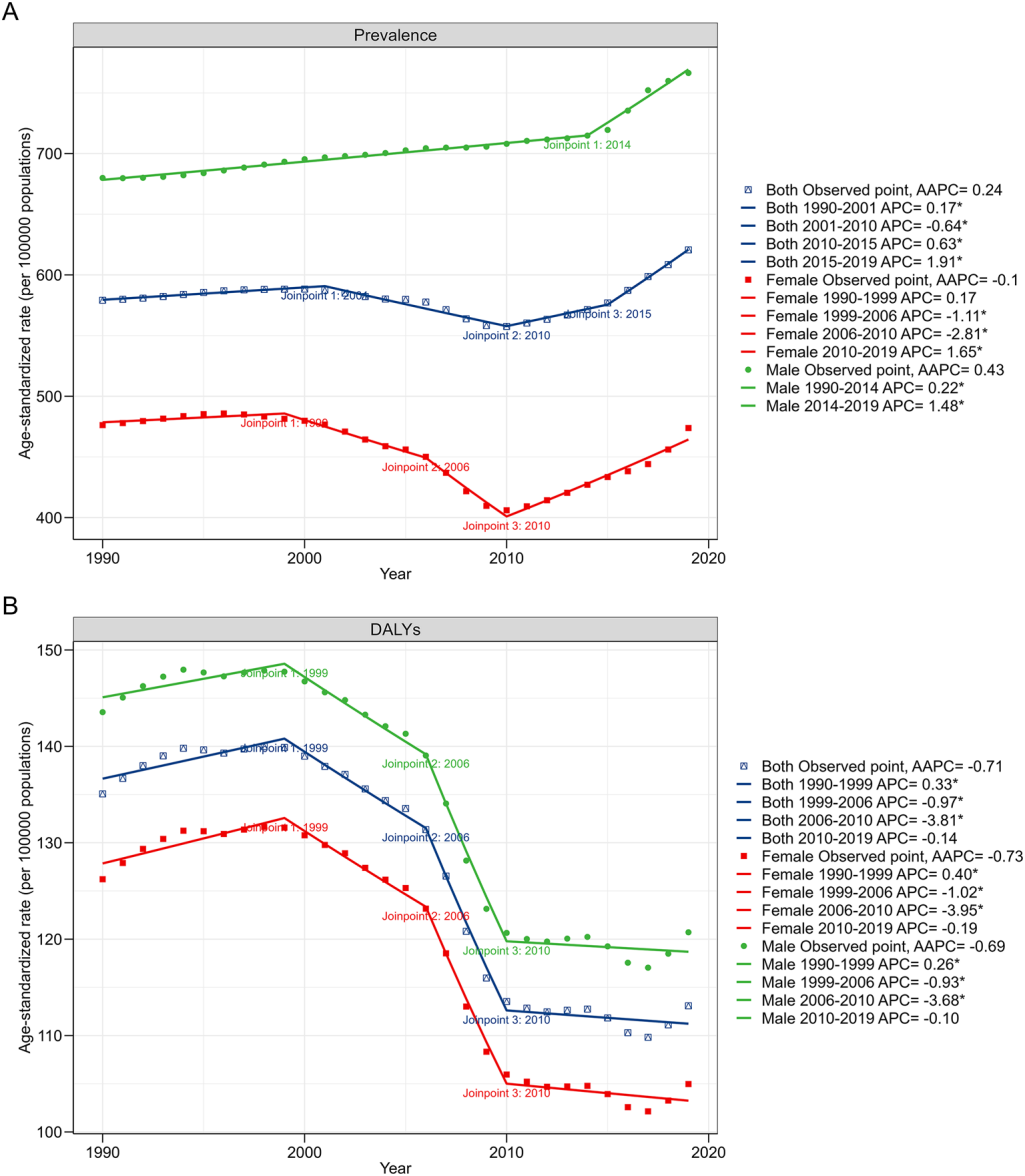
Results of Joinpoint regression analysis grouped by sex. (**A**) ASR of prevalence; (**B**) ASR of DALYs. *APC* annual percent change, *AAPC* average annual percent change.

Globally, the number of prevalent cases of syphilis was 30.91 million in 1990 and 49.71 million in 2019, with an increase of 60.83% from 1990 to 2019. (Table 1) The ASR of prevalence was stable from 1990 to 2019 (Fig. 1A), with an EAPC of 0.00 (95% CI − 0.10–0.11). The prevalence of ASR in 2019 was 766.41/100,000 for men and 473.81/100,000 for women (male/female = 1.62). The number of DALYs caused by syphilis was 7.36 million in 2019, a reduction of 16.38% compared to that in 1990 (8.80 million) (Table 2). The ASR of DALYs showed a decreasing trend from 1990 to 2019 (EAPC =  − 1.01; 95% CI − 1.19 to − 0.84) (Fig. 1B). A similar downward trend was observed in both men and women. The highest ASR prevalence was observed among those aged 15–49 years (1113.54/100,000), followed by the 50–69 years age group (364.20/100,000), and was the lowest in the < 14 years age group (31.53/100,000) (Fig. 1C). The highest ASR of DALYs was found in the < 14 years age group, which increased with increasing age. A reduction in DALYs over time was observed in all age-stratified subgroups (Fig. 1D). Joinpoint analysis was applied to divide the temporal trends into four time periods and estimate the APC by sex. The results showed that the ASR of prevalence increased slightly (AAPC = 0.24) between 1990 and 2019. The three periods of prevalence from 1990 to 2001, 2001 to 2010, and 2015 to 2019 showed an upward trend (APC > 0), whereas the period from 2001 to 2004 showed a downward trend (APC < 0). The upward trend in men was more pronounced than that in women (Fig. 2A). Joinpoint regression analysis revealed that the ASR of DALYs showed a downward trend in all four time periods (APC < 0 and AAPC < 0), and this trend was similar between men and women (Fig. 2B).

**Table 1:** ASR of the prevalence of syphilis between 1990 and 2019 and EAPC from 1990 to 2019.

Characteristics	1990 (95% UI)		2019 (95% UI)		Changes in Numbers (%)	EAPC in ASR (1990–2019, 95% CI)
	Number	ASR (per 100 000 population)	Number	ASR (per 100 000 population)		
Global	30,909,289 (23,606,899–41,440,264)	579.13 (447.51–769.06)	49,709,938 (38,256,228–66,047,911)	620.62 (476.62–822.87)	60.83	0.00 (− 0.10, 0.11)
Gender						
Male	18,032,512 (13,620,246–24,177,887)	679.9 (515.62–908.27)	31,048,278 (23,130,093–41,840,268)	766.41 (571.2–1040.20)	72.18	0.33 (0.27, 0.39)
Female	12,876,777 (9,969,777–17,263,089)	476.23 (368.95–631.97)	18,661,659 (14,933,934–24,108,788)	473.81 (380.04–611.98)	44.92	− 0.49 (− 0.68, − 0.29)
Age						
< 14	437,933 (184,779–792,611)	24.97 (10.53–45.19)	617,819 (298,498–1,059,971)	31.53 (15.23–54.09)	41.08	0.42 (0.29, 0.56)
15 − 49	27,761,124 (20,452,917–38,164,432)	1023.57 (754.11–1407.14)	43,819,118 (32,547,900–59,925,096)	1113.54 (827.12–1522.83)	57.84	0.03 (− 0.09, 0.14)
50 − 69	2,577,325 (1,722,579–3,802,850)	377.83 (252.53–557.49)	5,022,129 (3,334,840–7,438,333)	364.20 (241.84–539.42)	94.86	− 0.25 (− 0.30, − 0.19)
> 70	132,907 (95,896–179,870)	65.94 (47.58–89.24)	250,871 (176,282–349,338)	54.1 (38.02–75.34)	88.76	− 0.90 (− 0.97, − 0.83)
SDI						
Low	8,079,656 (6,393,435–10,409,046)	1809.13 (1443.52–2305.94)	14,821,701 (11,689,746–19,409,200)	1459.13 (1156.88–1869.7)	83.44	− 1.03 (− 1.13, − 0.93)
Low − middle	8,661,641 (6,499,190–11,829,996)	848.01 (643.2–1139.53)	14,708,818 (11,152,024–19,839,806)	803.79 (615.43–1073.8)	69.82	− 0.39 (− 0.50, − 0.28)
Middle	8,484,949 (6,387,862–11,546,903)	493.58 (375.37–656.85)	12,477,766 (9,378,910–16,753,626)	479.38 (359.61–647.92)	47.06	− 0.29 (− 0.44, − 0.15)
High − middle	3,533,335 (2,674,085–4,796,573)	289.18 (219.77–387.82)	5,082,442 (3,903,419–6,645,053)	322.47 (249.46–427.31)	43.84	0.12 (0.01, 0.23)
High	2,129,791 (1,621,007–2,837,606)	238.7 (181.59–316.91)	2,583,641 (1,971,555–3,407,502)	246.75 (185.96–333.47)	21.31	0.15 (0.10, 0.20)
Region						
East Asia	4,262,700 (3,163,913–5,871,930)	324.61 (242.67–438.61)	5,583,357 (4,157,561–7,572,992)	333.5 (248.18–456.69)	30.98	− 0.12 (− 0.26, 0.01)
South Asia	8,145,444 (6,056,688–11,294,682)	813.53 (606.19–1099.37)	12,893,135 (9,557,401–17,543,195)	679.56 (507.13–916.19)	58.29	− 0.89 (− 1.14, − 0.64)
Central Asia	121,181 (91,040–164,476)	184.68 (142.18–244.67)	190,314 (151,588–241,995)	191.09 (152.92–242.18)	57.05	− 0.01 (− 0.08, 0.06)
Southeast Asia	1,709,290 (1,254,273–2,352,819)	381.68 (284.26–519.99)	2,759,157 (2,089,572–3,718,042)	377.49 (285.84–507.9)	61.42	− 0.10 (− 0.15, − 0.05)
Eastern Europe	449,766 (343,175–588,631)	183.2 (140.51–240.38)	375,286 (287,612–498,169)	162.99 (125.43–214.6)	− 16.56	− 0.64 (− 0.75, − 0.54)
Western Europe	895,516 (676,859–1,195,754)	219.91 (165.64–296.3)	911,027 (695,362–1,213,509)	217.39 (162.43–296.29)	1.73	− 0.02 (− 0.05, 0.00)
Central Europe	194,064 (149,066–256,527)	150.92 (115.63–199.98)	183,632 (142,805–241,659)	153.58 (118.07–201.53)	− 5.38	0.11 (0.04, 0.18)
High -income Asia Pacific	498,039 (381,638–665,609)	261.52 (198.95–348.43)	500,764 (382,982–667,651)	270.42 (204.16–364.72)	0.55	0.27 (0.20, 0.35)
Oceania	106,681 (79,581–145,229)	1743.5 (1339.01–2322.94)	228,525 (167,178–317,316)	1721.86 (1284.46–2378.81)	114.21	− 0.62 (− 0.86, − 0.37)
Australasia	47,194 (35,403–64,184)	216.43 (163.08–294.78)	61,950 (46,668–83,397)	211.48 (158.41–287.1)	31.27	− 0.10 (− 0.13, − 0.07)
High-income North America	731,293 (554,399–965,993)	237.59 (181.36–313.43)	925,592 (711,765–1,206,547)	246.16 (186.91–328.76)	26.57	0.11 (− 0.09, 0.32)
Andean Latin America	266,275 (198,300–368,515)	759.5 (577.43–1026.19)	458,820 (357,373–596,179)	697.79 (545.06–903.63)	72.31	− 0.60 (− 0.71, − 0.48)
Central Latin America	666,064 (493,025–925,842)	437.07 (329.55–590.6)	1,099,152 (858,336–1,429,093)	416.88 (325.99–540.91)	65.02	− 0.14 (− 0.18, − 0.10)
Tropical Latin America	685,451 (506,563–964,040)	450.02 (337.18–619.2)	1,287,388 (1,059,276–1,567,635)	528.33 (434.41–645.78)	87.82	− 0.65 (− 1.51, 0.22)
Southern Latin America	206,026 (156,903–279,826)	420.09 (320.83–568.65)	336,183 (298,389–382,736)	485.29 (431.04–550.79)	63.18	0.34 (0.08, 0.60)
Caribbean	211,429 (158,416–292,317)	594.73 (452.48–806.69)	371,168 (322,827–434,450)	762.7 (664.79–891.58)	75.55	0.76 (0.64, 0.89)
Eastern Sub-Saharan Africa	4,392,799 (3,582,576–5,429,115)	2760.31 (2269.02–3384.02)	6,953,752 (5,699,833–8,748,090)	1893.65 (1546.59–2355.28)	58.30	− 1.79 (− 2.03, − 1.55)
Southern Sub-Saharan Africa	1,737,525 (1,379,608–2,214,748)	3399.31 (2703.24–4304.19)	2,064,080 (1,526,701–2,800,180)	2446.68 (1837.84–3278.58)	18.79	− 1.26 (− 1.84, − 0.68)
Western Sub-Saharan Africa	2,764,941 (2,105,880–3,717,996)	1722.37 (1327.3–2284.68)	6,222,789 (4,703,205–8,343,489)	1544.5 (1188.98–2040.18)	125.06	− 0.39 (− 0.45, − 0.32)
North Africa and Middle East	911,701 (670,883–1,256,460)	300.96 (226.45–407.08)	2,082,011 (1,542,894–2,839,126)	315.41 (237.13–426.3)	128.37	0.28 (0.06, 0.50)
Central Sub-Saharan Africa	1,905,910 (1,458,697–2,515,827)	4145.22 (3237.32–5378.77)	4,221,853 (3,277,797–5,580,811)	3612.56 (2816.27–4688.08)	121.51	− 0.77 (− 0.90, − 0.64)

*ASR* age-standardized rate, *EAPC* estimated annual percentage changes, *SDI* sociodemographic index.

**Table 2:** ASR of DALYs rate of syphilis between 1990 and 2019 and EAPC from 1990 to 2019.

Characteristics	1990 (95% UI)		2019 (95% UI)		Changes in Numbers (%)	EAPC in ASR (1990–2019, 95% CI)
	Number	ASR (per 100 000 population)	Number	ASR (per 100 000 population)		
Global	8,799,826 (2,941,190–18,551,504)	135.08 (45.61–284.1)	7,358,589 (2,580,712–15,037,475)	113.09 (39.49–231.38)	− 16.38	− 1.01 (− 1.19, − 0.84)
Gender						
Male	4,808,669 (1,619,308–10,225,722)	143.55 (49.12–304.07)	4,060,733 (1,426,881–8,349,353)	120.71 (42.23–248.61)	− 15.55	− 0.98 (− 1.15, − 0.82)
Female	3,991,157 (1,356,980–8,447,460)	126.22 (43.07–266.91)	3,297,856 (1,154,328–6,788,196)	104.97 (36.61–216.29)	− 17.37	− 1.05 (− 1.24, − 0.87)
Age						
< 14	8,685,362 (2,827,611–18,438,968)	495.18 (161.21–1051.27)	7,264,347 (2,492,518–14,941,417)	370.68 (127.19–762.42)	− 16.36	− 1.19 (− 1.32, − 1.06)
15 − 49	72,157 (51,182–87,594)	2.66 (1.89–3.23)	59,575 (44,703–76,397)	1.51 (1.14–1.94)	− 17.44	− 2.44 (− 2.62, − 2.27)
50 − 69	32,999 (20,516–44,301)	4.84 (3.01–6.49)	25,687 (18,195–31,868)	1.86 (1.32–2.31)	− 22.16	− 4.04 (− 4.35, − 3.73)
> 70	9309 (7282–11,255)	4.62 (3.61–5.58)	8980 (7369–10,940)	1.94 (1.59–2.36)	− 3.53	− 3.60 (− 3.92, − 3.27)
SDI						
Low	4,381,521 (1,480,768–9,208,549)	400.33 (139.67–834.95)	4,255,619 (1,525,621–8,683,202)	239.21 (86.63–486.2)	− 2.87	− 2.10 (− 2.24, − 1.95)
Low - middle	2,663,300 (863,448–5,722,160)	149.97 (50.18–320.24)	1,813,837 (604,887–3,835,753)	106.78 (35.61–225.77)	− 31.90	− 1.57 (− 1.84, − 1.31)
Middle	1,466,391 (500,363–3,142,534)	71.6 (24.73–152.86)	1,095,895 (363,169–2,336,659)	63.01 (20.64–134.88)	− 25.27	− 0.54 (− 0.58, − 0.49)
High -middle	252,899 (97,369–519,207)	25.07 (9.5–51.71)	162,157 (60,186–338,122)	20.47 (7.11–43.56)	− 35.88	− 0.75 (− 0.94, − 0.56)
High	27,430 (15,122–48,821)	4.15 (2.01–7.89)	20,916 (11,673–35,877)	3.14 (1.35–6.09)	− 23.75	− 0.31 (− 0.62, 0.00)
Region						
East Asia	405,562 (133,684–867,635)	33.79 (11.19–72.11)	175,055 (59,781–388,920)	22.3 (6.96–50.72)	− 56.84	− 0.76 (− 1.08, − 0.44)
South Asia	2,351,691 (718,322–5,148,186)	139.77 (43.82–303.9)	1,244,498 (397,901–2,719,777)	77.52 (24.7–169.58)	− 47.08	− 2.60 (− 3.17, − 2.03)
Central Asia	10,987 (5017–20,687)	12.81 (6.38–23.21)	10,489 (3479–21,228)	11.51 (3.81–23.28)	− 4.53	− 0.61 (− 0.94, − 0.28)
Southeast Asia	504,517 (155,372–1,148,083)	84.5 (26.57–191.44)	495,368 (155,108–1,088,499)	93.88 (29.32–206.71)	− 1.81	0.52 (0.31, 0.74)
Eastern Europe	7099 (4367–7848)	3.13 (2.1–3.43)	1523 (1263–2284)	0.88 (0.74–1.16)	− 78.55	− 8.12 (− 9.79, − 6.45)
Western Europe	8848 (5608–14,535)	2.98 (1.56–5.56)	5886 (2945–10,932)	2.3 (0.88–4.67)	− 33.48	0.30 (− 0.28, 0.89)
Central Europe	4059 (1801–8197)	4.62 (1.82–9.73)	1470 (681–2836)	2.39 (0.89–5.04)	− 63.78	− 2.76 (− 3.03, − 2.49)
High- income Asia Pacific	6845 (3059–13,730)	6.3 (2.35–13.53)	4842 (2336–9085)	5.87 (2.13–12.31)	− 29.26	0.23 (− 0.06, 0.52)
Oceania	55,513 (18,617–118,215)	526 (176.48–1119.12)	96,012 (32,103–194,984)	490.51 (165.24–994.81)	72.95	− 0.77 (− 1.02, − 0.52)
Australasia	180 (151–215)	0.96 (0.81–1.16)	90 (72–117)	0.26 (0.21–0.35)	− 50.00	− 3.44 (− 4.30, − 2.58)
High- income North America	5339 (4368–6521)	1.75 (1.45–2.12)	4884 (3798–6199)	0.99 (0.78–1.23)	− 8.52	− 2.23 (− 2.49, − 1.97)
Andean Latin America	103,822 (31,348–226,750)	181.46 (55.55–395.49)	50,568 (16,590–109,128)	80.07 (26.29–172.88)	− 51.29	− 4.01 (− 4.36, − 3.65)
Central Latin America	22,532 (16,506–32,296)	10.53 (8.01–14.66)	8905 (5938–13,809)	3.98 (2.58–6.28)	− 60.48	− 3.68 (− 3.90, − 3.46)
Tropical Latin America	59,779 (44,462–81,797)	35.41 (26.4–48.32)	39,815 (25,768–61,285)	24.91 (15.9–38.78)	− 33.40	− 0.80 (− 1.42, − 0.18)
Southern Latin America	6180 (5380–7084)	12.38 (10.78–14.18)	1916 (1443–2507)	3.72 (2.72–4.99)	− 69.00	− 3.85 (− 4.47, − 3.22)
Caribbean	71,766 (24,598–151,553)	167.13 (58.35–351.84)	85,921 (31,297–176,153)	218.89 (79.4–449.64)	19.72	0.64 (0.30, 0.99)
Eastern Sub-Saharan Africa	2,657,139 (920,017–5,531,372)	650.73 (232.88–1341.61)	2,267,482 (835,678–4,576,728)	342.64 (128.09–687.87)	− 14.66	− 2.55 (− 2.81, − 2.28)
Southern Sub-Saharan Africa	394,664 (139,024–800,770)	543.22 (195.26–1096.38)	260,174 (90,526–527,492)	326.64 (113.78–661.99)	− 34.08	− 1.78 (− 2.20, − 1.35)
Western Sub-Saharan Africa	1,103,869 (355,226–2,401,585)	265.1 (86.97–574.72)	1,619,790 (535,869–3,389,369)	209.12 (69.38–436.87)	46.74	− 0.66 (− 0.85, − 0.47)
North Africa and Middle East	234,410 (71,522–540,526)	42.21 (13.03–97.07)	226,059 (69,409–494,687)	38.72 (11.9–84.73)	− 3.56	− 1.35 (− 1.79, − 0.91)
Central Sub–Saharan Africa	785,024 (268,863–1,596,569)	635.77 (220.17–1285.86)	757,842 (263,061–1,563,105)	360.36 (125.48–740.47)	− 3.46	− 2.67 (− 2.99, − 2.35)

*DALYs* disability‐adjusted life years, *ASR* age-standardized rate, *EAPC* estimated annual percentage changes, *SDI* sociodemographic index.

### Prevalence in different regions

The ASR of syphilis prevalence has been investigated in different regions worldwide and is reported to be negatively associated with the SDI level. In 2019, the highest ASR of prevalence was observed in the low SDI regions (1459.13/100,000) and lowest in the high SDI regions (246.75/100,000) (Fig. 1E). There was an increasing trend in the ASR of prevalence in high SDI regions (EAPC: 0.15, 95% CI 0.10–0.20) and high-middle SDI regions (EAPC: 0.12, 95% CI 0.01–0.23), whereas the incidence decreased in low SDI regions (EAPC: − 1.03, − 1.13 to − 0.93).

**Figure 3:**
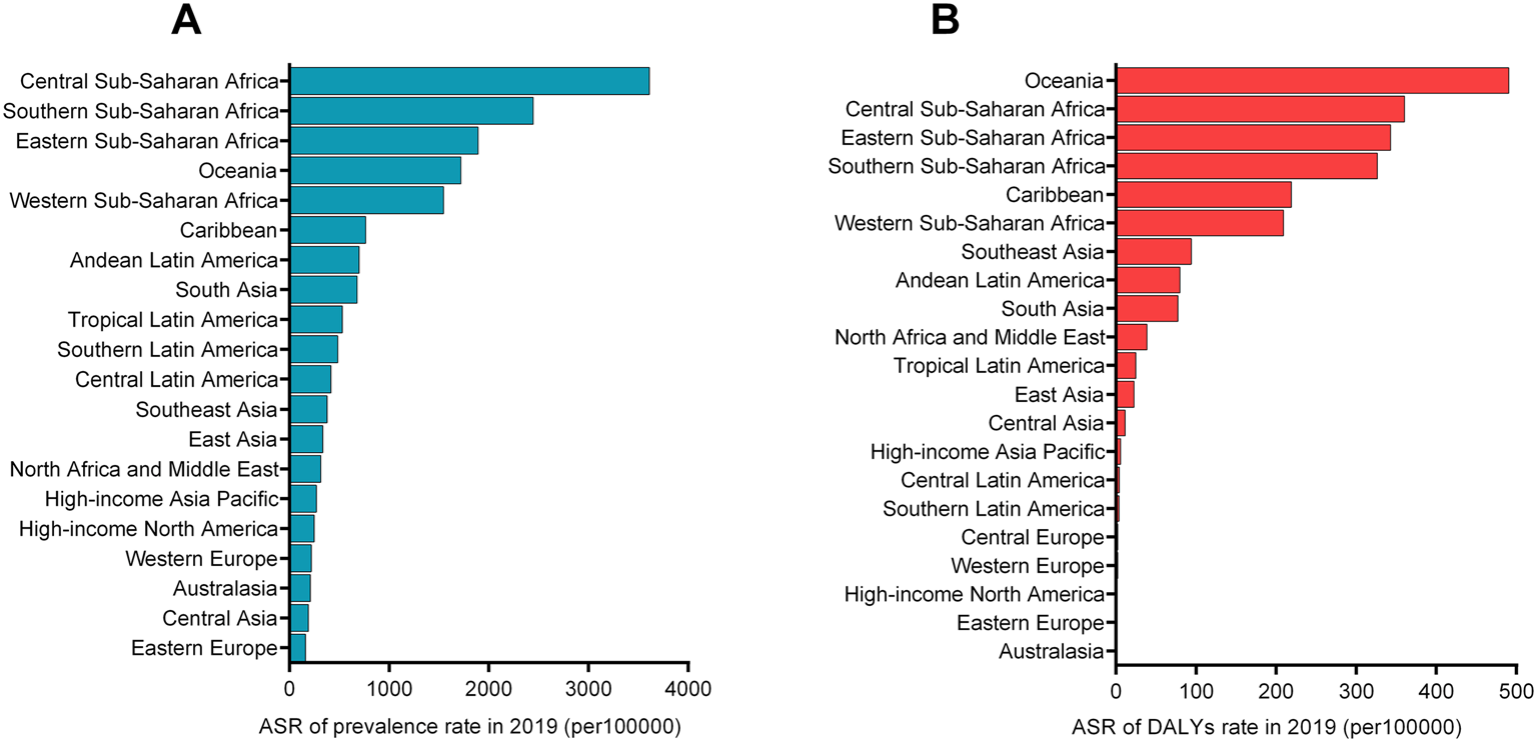
ASR of syphilis in 21 geographical regions in 2019. (**A**) ASR of prevalence; (**B**) ASR of DALYs. *DALYs* disability‐adjusted life years, *ASR* age-standardized rate.

**Figure 4:**
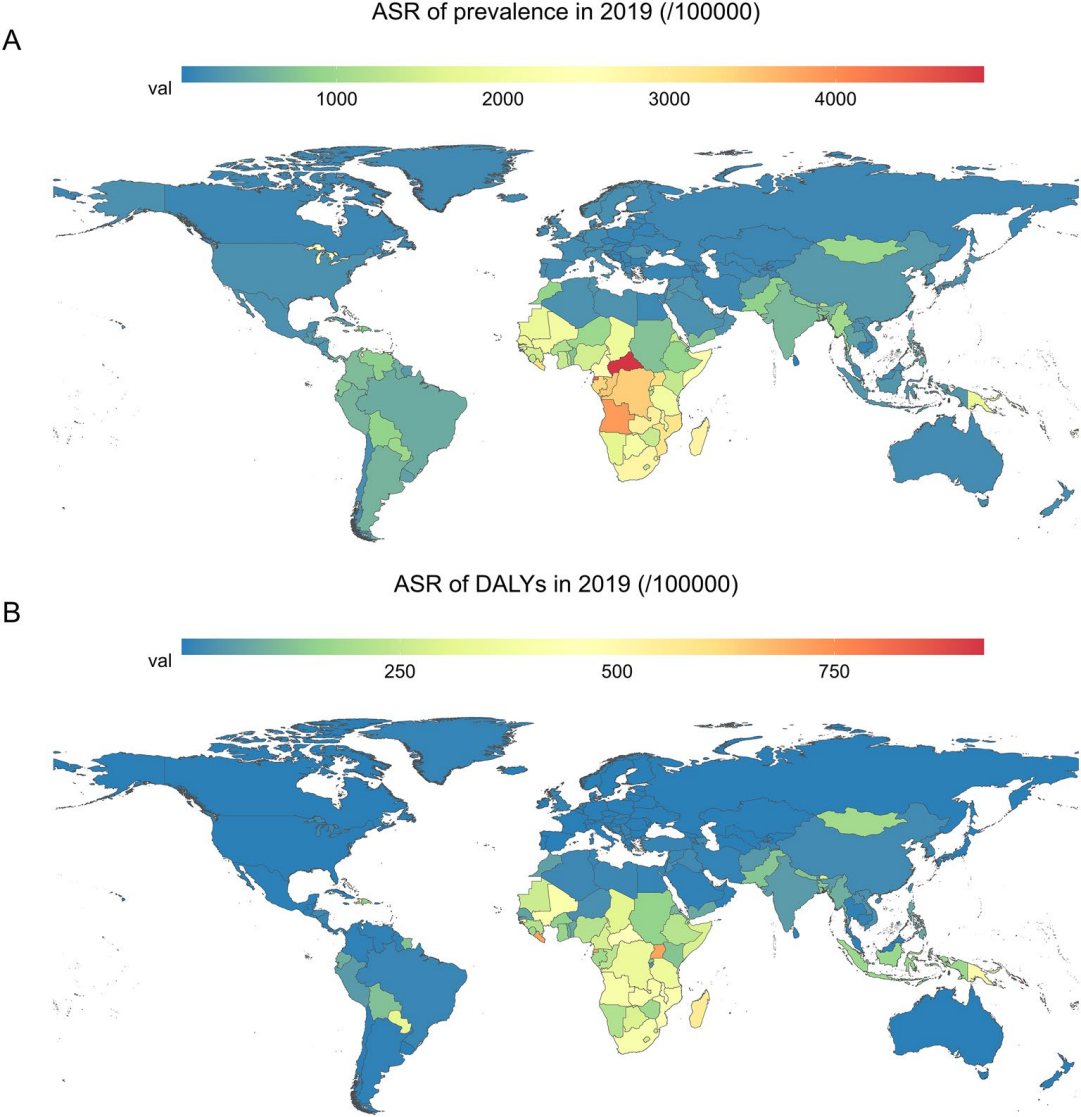
ASR of syphilis in 204 countries or territories in 2019. (**A**) ASR of prevalence; (**B**) ASR of DALYs. The world map chart was built using the R statistical software (version 4.2.2). Prevalence and DALYs of syphilis worldwide was mapped using the “sf” R package and “.shp file”. *DALYs* disability‐adjusted life years, *ASR* age-standardized rate.

The highest observed ASR of prevalence in 2019 was in Central Sub-Saharan Africa, followed by Southern Sub-Saharan Africa and Eastern Sub-Saharan Africa (Fig. 3A). In addition, among the 204 countries or territories, the Central African Republic (4883.08/100,000), Equatorial Guinea (4429.62/100,000), and Angola (3892.36/100,000) exhibited the highest ASR of prevalence in 2019 (Supplementary Table 1, Fig. 4A). However, the ASR of prevalence decreased significantly over time among the three regions. The three countries and territories with the highest increase in ASR of prevalence were Greece, Mongolia, and Paraguay, with EAPCs of 3.59, 3.31, and 1.38, respectively.

### DALYs in different regions

The highest ASR of DALYs occurred in low-SDI regions (239.21/100,000) and the lowest occurred in high-SDI regions (3.14/100,000). Generally, the ASR of DALYs decreased as the SDI increased. (Fig. 1F). We also found decreasing trends of DALYs in all SDI areas, particularly in low SDI areas (EAPC =  − 2.10; 95% CI − 2.24 to − 1.95). In 2019, the highest observed ASR of DALYs was in Oceania, followed by Central Sub-Saharan Africa and Eastern Sub-Saharan Africa (Fig. 3B). The ASR of DALYs showed decreasing trends in most regions, particularly Eastern Europe (EAPC =  − 8.12; 95% CI − 9.79 to − 6.45) and Southern Latin America (EAPC =  − 3.85; 95% CI − 4.47 to − 3.22). An upward trend in the ASR of DALYs was observed exclusively in the Caribbean and Southeast Asia, with EAPCs of 0.64 (95% CI 0.30–0.99) and 0.52 (95% CI 0.31–0.74), respectively.

The top three countries and territories with the highest ASR of DALYs due to syphilis were the Solomon Islands (920.93/100,000), Equatorial Guinea (727.02/100,000), and Liberia (717.24/100,000), while those with the lowest were Slovenia, Lithuania, and Hungary (Fig. 4B). The details are listed in Supplementary Table 2. Overall, the ASR of DALYs exhibited a predominantly declining trend across most regions worldwide. Additionally, Turkmenistan (EAPC =  − 9.58 95% CI − 11.48 to − 7.68) and Panama (EAPC = 3.51, 95% CI 2.46–4.56) exhibited the most significant downward and upward trend in ASR of DALYs, respectively.

## Discussion

This study provides a comprehensive estimation of the global prevalence of syphilis and its associated DALYs, over the past three decades. The main findings were as follows: (1) The ASR of the prevalence of syphilis remained stable from 1990 to 2019, whereas the ASR of the DALYs due to syphilis decreased globally. (2) The highest ASR of the DALYs was observed among individuals younger than 14 years. (3) The burden of syphilis decreased with an increase in the SDI. Solomon Islands, Equatorial Guinea, and Liberia exhibited the highest age-standardized DALYs rates in 2019 among 204 countries or territories investigated.

Several regional studies have provided evidence that affirms the persistently high prevalence of syphilis^7–9,20^. The reason may be due to the development of diagnostic capacity in the countries studied, which has led to an increase in the number of declared cases. Additionally, the increased numbers of men engaging in same-sex sexual activity^21^, and the patterns of sexual behavior among adolescents may play a relatively crucial role^22^. Furthermore, an increase in the frequency of travel may be attributed to the transmission of syphilis^23^. While pre-exposure and post-exposure prevention measures are available to mitigate the risk of STIs^24,25^, insufficient awareness of syphilis may be a probable reason for its persistence in certain regions^26^. The use of condoms is the most economical and simple way to prevent the spread of syphilis and other STIs^27^. Several governments have implemented a series of measures and efforts to promote the use of condoms and prevent the spread of STIs^28^. For instance, government initiatives provide free or affordable condoms, while family planning and healthcare organizations distribute free condoms to individuals, concurrently promoting safe sex education. Volunteers are additionally mobilized within the community to raise awareness and educate people about condoms and safe sex practices. Furthermore, governments have actively promoted the implementation of comprehensive sex education programs in schools and communities. These initiatives aim to educate young individuals and adults about contraception, safe sexual practices, and the importance of understanding their own physical and sexual behaviors. The objective is to help individuals develop realistic reproductive health plans^29,30^.

Although syphilis has a high prevalence, the ASR of the DALYs has decreased over the past three decades. This may have been due to the effectiveness of the current therapy. Furthermore, the declining trend in syphilis cases may be attributed to the expanding diagnostic capabilities and improved access to health services, particularly in terms of antimicrobial agent treatment. Antibiotics are the most effective treatment for syphilis^31^ and can significantly reduce the morbidity and mortality of syphilis in humans^32^. However, antimicrobial resistance has increased worldwide as a consequence of the widespread use of antimicrobial agents. At present, many countries are experiencing an increase in antibiotic resistance, which poses a growing threat to the burden of syphilis^33,34^. Appropriate use of antibiotics is indispensable to minimize the burden of antibiotic resistance in syphilis^35^. In addition, there is an urgent need for the development of novel antibiotics^36^. Finally, syphilis has a wide variety of clinical manifestations and can be easily misdiagnosed if the specific antibody is not tested^37^. In addition to causing damage to the genitourinary system, syphilis has to potential to harm other organs, including the cardiovascular, nervous, and ocular systems^38,39^. If left untreated, syphilis can pose life-threatening risks.

Additionally, we observed a paradoxical phenomenon, in which the age group aged under 14 years exhibited the lowest prevalence but the highest rate of DALYs. This indicates that early exposure to syphilis can lead to adverse outcomes. Therefore, there is a critical need for sexual protection for syphilis among younger individuals^40^. Another important reason for the high burden of DALYs in this age group may be the vertical transmission of syphilis, which may contribute to poorer outcomes in younger individuals^41^. Congenital syphilis due to vertical transmission can cause neonatal death, as well as cutaneous and visceral manifestations. Early diagnosis and treatment are effective measures to reduce prevalence and mortality^42^. Therefore, it is crucial to strengthen the existing screening practices for pregnant women and establish thorough follow-up evaluations for women who have contracted syphilis^43,44^.

Another important finding of our study was that the burden of syphilis decreased with the increase in SDI level. This is consistent with the cross-sectional study by Costa et al., which showed a significant association between family income and STIs^45^. Additionally, the present study highlights the substantial disease burden of STIs in developing countries, particularly in Sub-Saharan Africa, Oceania, and the Caribbean. Hence, extensive strategies for the effective control and management of syphilis should be extensively implemented in developing countries for screening, education, and treatment^46,47^. The Solomon Islands, Equatorial Guinea, and Liberia were among the less developed countries in the world^48,49^ that exhibited the highest ASR of DALYs.

## Limitations

The study is subject to certain inherent limitations. First, data from the GBD database primarily focuses on economically developed countries, and there may be a lack of comprehensive health data for certain poorer or developing countries. As a result, the health situation in these regions may not be fully represented^50^. Furthermore, the collection and update of data may have certain delays, particularly during emergencies or large-scale outbreaks. As a result, there could be some lag in the availability of updated data. There are several suggestions for future research in this field. With the advancement of technology and the development of data collection methods, more accurate data can be obtained by enhancing the speed of data updates. Furthermore conducting data analysis from multiple perspectives through the integration of different data sources, analysis methods, and disease classification standards can improve the accuracy and precision of the data.

## Conclusion

The ASR of the prevalence of syphilis remained persistently high from 1990 to 2019 worldwide, with no significant changes observed. Conversely, the ASR of DALYs attributed to syphilis exhibited a decrease over the same period. There was an inverse relationship between the syphilis burden and SDI levels. Individuals under the age of 14 and regions with a low SDI require greater attention in addressing the burden of syphilis.

## Supplementary Information

Supplementary Table 1.

Supplementary Table 2.

## Data Availability

The data used in this study were publicly accessed. To download the dataset, please visit the Global Health Data Exchange at http://ghdx.healthdata.org/gbd-results-tool.

## Abbreviations

STIs: Sexually transmitted infections
DALYs: Disability‐adjusted life years
ASR: Age-standardized rate
EAPC: Estimated annual percentage changes
GBD: Global burden of diseases
SDI: Sociodemographic index
UI: Uncertainty intervals
CI: Confidence interval
