# Supplementary material for: Evaluating the global, regional, and national impact of syphilis: results from the global burden of disease study 2019
Source: Sci Rep. 2023 Jul 14;13:11386. doi: 10.1038/s41598-023-38294-4 (PMC10349077; doi:10.1038/s41598-023-38294-4)
Supplement: Supplementary file 1 — Supplementary Table 1. [file 41598_2023_38294_MOESM1_ESM.doc]

Supplementary Table 1 Prevalence of syphilis in 204 countries and territories in 1990 and 2019 and EAPC from 1990 to 2019

| 204 countries or territories | ASR of Prevalence rate (1990) | ASR of Prevalence rate (2019) | EAPC in ASR (1990–2019, 95% CI) |
| --- | --- | --- | --- |
| Afghanistan | 362.52 (268.77–496.93) | 384.55 (288.97–527.4) | 0.12 (0.05, 0.18) |
| Albania | 131.35 (100.95–175.31) | 127.01 (95.99–171.41) | −0.12 (−0.17, −0.07) |
| Algeria | 282.91 (212.29–381.47) | 263.15 (198.98–347.43) | −0.30 (−0.48, −0.13) |
| American Samoa | 1068.08 (804.18–1453.81) | 1055.97 (787.42–1437.48) | −0.04 (−0.07, −0.01) |
| Andorra | 220.37 (164.32–298.28) | 212.42 (158.49–289.58) | −0.14 (−0.18, −0.11) |
| Angola | 4481.53 (3455.55–5874.97) | 3892.36 (2949.3–5249.4) | −0.65 (−0.71, −0.58) |
| Antigua and Barbuda | 436.15 (332.36–593.2) | 436.35 (334.04–585.08) | −0.08 (−0.14, −0.02) |
| Argentina | 474.92 (361.22–642.74) | 589.68 (527.23–655.98) | 0.67 (0.24, 1.10) |
| Armenia | 166.84 (128.72–216.47) | 142.11 (108.04–190.97) | −0.88 (−0.99, −0.76) |
| Australia | 213.29 (160.47–291.24) | 209.04 (156.23–284.78) | −0.09 (−0.12, −0.06) |
| Austria | 212.34 (157.9–286.03) | 209.69 (155.31–284.51) | −0.05 (−0.08, −0.02) |
| Azerbaijan | 190.59 (145.76–252.34) | 168.46 (128.07–226.77) | −0.59 (−0.69, −0.48) |
| Bahamas | 627.35 (468.26–861.91) | 662.39 (542.12–818.32) | 0.56 (0.41, 0.70) |
| Bahrain | 321.18 (241.71–434.32) | 323.02 (240.74–435.53) | 0.15 (0.10, 0.20) |
| Bangladesh | 933.87 (705.86–1252.88) | 745.44 (563.09–1000.24) | −1.40 (−1.62, −1.18) |
| Barbados | 355.01 (272.21–467) | 351.8 (268.56–467.51) | −0.04 (−0.05, −0.02) |
| Belarus | 139.63 (108.03–182.47) | 127.14 (99.01–165.45) | −0.50 (−0.58, −0.42) |
| Belgium | 213.12 (158.67–287.94) | 208.49 (156.12–283.77) | −0.11 (−0.14, −0.08) |
| Belize | 431.8 (330.29–579.74) | 427.1 (324.08–576.19) | −0.20 (−0.26, −0.13) |
| Benin | 860.04 (649.93–1130.32) | 736.85 (557.09–990.31) | −1.62 (−2.09, −1.14) |
| Bermuda | 497.39 (378–663.13) | 494.17 (374.78–668.21) | 0.00 (−0.05, 0.04) |
| Bhutan | 1210.19 (904.94–1673.1) | 1190.62 (882.63–1644.92) | −0.54 (−0.73, −0.36) |
| Bolivia (Plurinational State of) | 949.31 (720.83–1294.38) | 889.53 (727.48–1096.15) | −0.76 (−0.94, −0.57) |
| Bosnia and Herzegovina | 127.86 (96.9–169.82) | 126.74 (95.32–170.35) | −0.01 (−0.04, 0.03) |
| Botswana | 3390.29 (2884.24–3995.6) | 2076.4 (1554.54–2855.77) | −2.51 (−3.23, −1.80) |
| Brazil | 441.6 (330.69–607.85) | 513.31 (422.95–623.71) | −0.82 (−1.74, 0.10) |
| Brunei Darussalam | 256.74 (194.98–341.62) | 251.74 (190.5–338.72) | −0.02 (−0.08, 0.04) |
| Bulgaria | 115.07 (86.98–153.04) | 114.29 (87.42–153.82) | −0.02 (−0.05, 0.01) |
| Burkina Faso | 1155.63 (875.93–1541.87) | 1151.25 (917.22–1435.53) | 0.27 (0.15, 0.39) |
| Burundi | 1458.61 (1121.96–1891.46) | 1206.32 (918.45–1570.78) | −1.05 (−1.43, −0.67) |
| Cabo Verde | 1847.27 (1389.44–2491.82) | 2045.5 (1514.88–2745.51) | 0.32 (0.25, 0.40) |
| Cambodia | 133.55 (100.03–179.22) | 128.65 (96.78–172.02) | −0.51 (−0.68, −0.33) |
| Cameroon | 3598.23 (2824.72–4500.62) | 2555.98 (1923.74–3429.36) | −1.14 (−1.38, −0.91) |
| Canada | 166.22 (126.52–220.95) | 163.85 (124.26–216.06) | −0.11 (−0.25, 0.02) |
| Central African Republic | 4591.29 (3531.28–5963.06) | 4883.08 (3727.95–6065.85) | −0.28 (−0.40, −0.16) |
| Chad | 2023.24 (1537.24–2738.75) | 1884.42 (1416.67–2565.62) | −0.55 (−0.76, −0.34) |
| Chile | 288.97 (216.02–388.89) | 239.62 (191.2–316.26) | −1.37 (−1.79, −0.95) |
| China | 325.09 (242.71–439.52) | 334.11 (248.55–457.3) | −0.13 (−0.27, 0.01) |
| Colombia | 690.14 (519.71–936.33) | 669.65 (533.79–866.62) | 0.00 (−0.04, 0.03) |
| Comoros | 2203.16 (1690.99–2912.24) | 1522.12 (1171.1–2020.27) | −1.81 (−2.06, −1.57) |
| Congo | 3751.25 (2869.95–4902.11) | 3488.46 (2777.12–4433.68) | −0.38 (−0.43, −0.33) |
| Cook Islands | 1058.57 (792.14–1445.17) | 1045.27 (782.47–1424.09) | −0.03 (−0.06, 0.00) |
| Costa Rica | 380.35 (290.17–509.26) | 377.3 (285.36–503.29) | −0.02 (−0.04, 0.01) |
| Côte d'Ivoire | 1615.61 (1281.17–2052.89) | 1344.74 (1008.49–1800.03) | −0.53 (−0.66, −0.39) |
| Croatia | 126.37 (97.46–169.47) | 125.96 (96.05–169.73) | 0.00 (−0.03, 0.03) |
| Cuba | 431.94 (326.17–594.94) | 404.79 (314.72–530.09) | −0.54 (−0.85, −0.24) |
| Cyprus | 212.87 (160.11–287.53) | 207.82 (155.23–286.49) | −0.10 (−0.12, −0.09) |
| Czechia | 125.77 (96.34–167.7) | 126.76 (96.49–169.24) | 0.05 (0.02, 0.08) |
| Democratic People's Republic of Korea | 302.76 (228.48–411.92) | 307.66 (231.47–419) | 0.02 (0.00, 0.03) |
| Democratic Republic of the Congo | 4038.73 (3138.11–5231.24) | 3442.49 (2694.02–4453.45) | −0.87 (−1.05, −0.69) |
| Denmark | 197.67 (147.07–265.7) | 193.78 (147.16–257.93) | −0.17 (−0.21, −0.12) |
| Djibouti | 726.23 (563.61–950) | 676.18 (516.36–890.66) | −0.27 (−0.46, −0.08) |
| Dominica | 836.96 (627.82–1149.54) | 835.03 (621.94–1156.28) | 0.20 (0.11, 0.28) |
| Dominican Republic | 639.78 (482.74–875.28) | 759.49 (640.15–905.59) | 0.21 (0.01, 0.41) |
| Ecuador | 776.28 (589.64–1059.07) | 763.53 (579.88–1031.43) | −0.05 (−0.08, −0.03) |
| Egypt | 172.18 (130.08–231.64) | 170.31 (127.25–228.47) | 0.08 (0.00, 0.17) |
| El Salvador | 310.64 (237.6–415.33) | 288.56 (219.66–385.51) | −0.36 (−0.41, −0.30) |
| Equatorial Guinea | 4595.08 (3544.8–6018.22) | 4429.62 (3657.69–5430.8) | −0.27 (−0.45, −0.09) |
| Eritrea | 1692.92 (1299.89–2227.01) | 1364.85 (1037.23–1804.8) | −0.91 (−0.99, −0.82) |
| Estonia | 152.57 (118.21–201.07) | 147.02 (112.67–197.36) | −0.14 (−0.25, −0.03) |
| Eswatini | 2794.46 (2027.55–3594.81) | 1632.22 (1257.5–2127.67) | −1.58 (−1.81, −1.35) |
| Ethiopia | 1919.42 (1419.07–2665.01) | 929.6 (675.44–1298.36) | −2.93 (−3.29, −2.56) |
| Fiji | 1104.44 (832.92–1502.14) | 1086.33 (818.42–1466.07) | −0.06 (−0.11, 0.00) |
| Finland | 212.54 (158–289.95) | 209.92 (155.98–284.9) | −0.05 (−0.08, −0.01) |
| France | 209.31 (155.92–284.36) | 206.8 (154.44–280.27) | −0.06 (−0.08, −0.03) |
| Gabon | 4091.96 (3236.29–5220.78) | 3359.29 (2594.21–4438.21) | −1.24 (−1.45, −1.03) |
| Gambia | 1447.64 (1113.86–1944.3) | 1369.22 (1019.31–1854.97) | −0.59 (−0.75, −0.43) |
| Georgia | 191.07 (146.4–254.35) | 182.17 (146.92–230.72) | −0.72 (−0.94, −0.50) |
| Germany | 205.72 (153.9–275.34) | 202.74 (151.37–279.76) | −0.11 (−0.16, −0.07) |
| Ghana | 1503.66 (1139.98–2031.36) | 1376.93 (1123.45–1696.46) | −0.48 (−0.60, −0.37) |
| Greece | 131.32 (116.13–148.78) | 211.25 (157.24–286.23) | 3.59 (2.70, 4.49) |
| Greenland | 200.49 (152.5–263.49) | 190.8 (146.6–253.56) | −0.14 (−0.17, −0.10) |
| Grenada | 757.34 (572.62–1040.77) | 874.05 (650.8–1138.99) | 0.68 (0.52, 0.85) |
| Guam | 1048.4 (789.48–1429.28) | 1050.95 (790.97–1444.6) | 0.02 (−0.02, 0.05) |
| Guatemala | 368.26 (283.31–490.44) | 349.46 (268.15–463.35) | −0.38 (−0.45, −0.30) |
| Guinea | 1726.2 (1317.69–2299.16) | 1481.26 (1152.05–1916.91) | −0.77 (−1.02, −0.51) |
| Guinea−Bissau | 2771.11 (2111.63–3637.43) | 1910.76 (1440.45–2533.93) | −1.88 (−2.62, −1.14) |
| Guyana | 555.55 (424.6–746.36) | 543.73 (413.19–733.43) | −0.09 (−0.14, −0.05) |
| Haiti | 999.75 (749.85–1363.19) | 1253.03 (1157.58–1369.76) | 0.57 (0.23, 0.91) |
| Honduras | 328.18 (252.26–437.41) | 297.48 (229.46–393.24) | −0.58 (−0.68, −0.47) |
| Hungary | 130.08 (99.19–173.18) | 131.46 (100.29–173.29) | 0.08 (0.05, 0.11) |
| Iceland | 213.44 (159.19–290) | 209.85 (156.76–288.88) | −0.08 (−0.11, −0.05) |
| India | 792.85 (588.42–1071.3) | 641.85 (480.01–861.23) | −1.04 (−1.36, −0.73) |
| Indonesia | 333.1 (247.48–454.04) | 311.68 (230.53–424.32) | −0.29 (−0.32, −0.27) |
| Iran (Islamic Republic of) | 153.52 (111.57–215.26) | 162.59 (119.03–225.91) | 0.21 (0.05, 0.37) |
| Iraq | 280.63 (208.64–378.6) | 275.54 (204.22–371.91) | −0.08 (−0.09, −0.07) |
| Ireland | 207.73 (155.2–281.54) | 205.93 (153.74–285.04) | −0.04 (−0.06, −0.02) |
| Israel | 206.15 (154.8–281.13) | 207.79 (153.74–283.79) | 0.03 (0.00, 0.06) |
| Italy | 247.41 (183.54–335.98) | 236.19 (175.8–320.2) | −0.25 (−0.29, −0.21) |
| Jamaica | 664.98 (504.98–913.13) | 656.54 (540.45–801.69) | 0.78 (0.52, 1.04) |
| Japan | 274.36 (206.93–369.37) | 273.22 (205.99–369.78) | 0.01 (−0.04, 0.06) |
| Jordan | 358.14 (267.34–488.69) | 362.54 (269.56–492.24) | 0.05 (−0.02, 0.11) |
| Kazakhstan | 165.26 (126.49–221.22) | 156.2 (121.45–202.26) | −0.30 (−0.39, −0.21) |
| Kenya | 1874.68 (1426.82–2503.9) | 1342.45 (1015.67–1780.77) | −1.74 (−2.12, −1.36) |
| Kiribati | 1484.5 (1110.97–1995.27) | 1524.67 (1172.43–1978.39) | 0.13 (0.04, 0.22) |
| Kuwait | 293.97 (221.67–399.52) | 271.36 (200.87–366.56) | −0.26 (−0.32, −0.20) |
| Kyrgyzstan | 148.21 (112.74–199.08) | 134.15 (103.34–175.02) | −0.58 (−0.72, −0.44) |
| Lao People's Democratic Republic | 263.92 (196.52–362.17) | 263.19 (195.75–356.45) | −0.03 (−0.06, −0.01) |
| Latvia | 159.23 (124.15–206.18) | 145.5 (111.28–194.84) | −0.46 (−0.56, −0.35) |
| Lebanon | 272.55 (201.51–369.79) | 273.77 (201.29–373.75) | 0.00 (−0.04, 0.04) |
| Lesotho | 1847.15 (1383.39–2496.35) | 1324.7 (998.63–1781.94) | −0.92 (−1.08, −0.77) |
| Liberia | 3067.86 (2279.04–4086.83) | 3262.59 (2898.51–3697.22) | 0.44 (0.15, 0.72) |
| Libya | 284.87 (213.71–381.49) | 276.11 (207.23–377.08) | −0.06 (−0.11, −0.02) |
| Lithuania | 145.94 (112.02–194.58) | 141.44 (110.83–182.13) | −0.15 (−0.20, −0.10) |
| Luxembourg | 224.08 (168.12–300.9) | 212.64 (159.47–291.21) | −0.25 (−0.30, −0.19) |
| Madagascar | 3312.22 (2527.3–4286.06) | 2820.15 (2354.06–3376.93) | −1.14 (−1.38, −0.90) |
| Malawi | 2431.07 (2083.67–2833.55) | 2173.81 (1726.18–2759.57) | −1.03 (−1.33, −0.73) |
| Malaysia | 220.57 (167.5–292.59) | 220.84 (164.01–299.7) | −0.04 (−0.09, 0.02) |
| Maldives | 241.92 (183.96–325.09) | 273.88 (200.95–377.18) | 0.38 (0.20, 0.57) |
| Mali | 1968.07 (1480.58–2641.49) | 1985.3 (1532.23–2500.21) | 0.30 (0.17, 0.42) |
| Malta | 203.93 (149.4–277.04) | 206.91 (155.42–282.21) | 0.03 (0.01, 0.06) |
| Marshall Islands | 1092.4 (825.08–1480.93) | 1164.42 (897.03–1534.42) | 0.49 (−0.03, 1.02) |
| Mauritania | 2068.85 (1561.77–2762.72) | 1804.54 (1350.33–2433.98) | −0.46 (−0.54, −0.37) |
| Mauritius | 337 (254.66–458.22) | 398.16 (296.63–517.31) | 0.72 (0.54, 0.89) |
| Mexico | 302.51 (229.25–409.62) | 253.92 (193.81–341.22) | −0.87 (−1.06, −0.68) |
| Micronesia (Federated States of) | 1384.48 (1042.12–1895.67) | 1461 (1137.52–1859.21) | 0.47 (0.32, 0.62) |
| Monaco | 209.71 (155.16–286.98) | 208.33 (154.99–286.43) | −0.09 (−0.11, −0.06) |
| Mongolia | 449.06 (321.76–617.42) | 975.3 (871.12–1055.48) | 3.31 (2.69, 3.93) |
| Montenegro | 128.8 (97.93–173.85) | 128.88 (97.64–171.69) | 0.00 (−0.03, 0.02) |
| Morocco | 890.29 (648.42–1243.07) | 897.41 (656.76–1221.7) | 1.35 (0.52, 2.19) |
| Mozambique | 5944.38 (4876.32–7016.55) | 3162.41 (2582.91–3870.4) | −2.31 (−2.62, −2.01) |
| Myanmar | 948.44 (681.07–1339.35) | 1023.43 (783.78–1335.93) | 0.59 (0.28, 0.90) |
| Namibia | 1981.4 (1505.33–2636.01) | 1673.53 (1268.78–2259.22) | −1.56 (−2.08, −1.04) |
| Nauru | 1082.39 (820.08–1486.07) | 1070.95 (795.13–1464.91) | −0.04 (−0.06, −0.03) |
| Nepal | 870.87 (661.31–1174.08) | 811.51 (604.79–1115.21) | −0.31 (−0.35, −0.26) |
| Netherlands | 212.46 (156.97–287.47) | 208.47 (156.44–286.64) | −0.13 (−0.16, −0.09) |
| New Zealand | 232.6 (172.73–314.42) | 226.9 (168.34–310.4) | −0.12 (−0.16, −0.07) |
| Nicaragua | 334.67 (259.13–447.45) | 292.77 (226.58–382.07) | −0.78 (−1.02, −0.53) |
| Niger | 1067.24 (800.04–1433.51) | 976.48 (727.4–1314.62) | −0.45 (−0.51, −0.39) |
| Nigeria | 1638.76 (1228.78–2201.74) | 1484.45 (1108.5–2020.5) | −0.28 (−0.38, −0.18) |
| Niue | 1055.41 (791.28–1443.59) | 1058.23 (787.72–1442.59) | 0.01 (−0.01, 0.03) |
| North Macedonia | 126.37 (96.37–168.14) | 128.05 (97.28–173.63) | 0.06 (0.03, 0.09) |
| Northern Mariana Islands | 1134.57 (860–1516.65) | 1090.34 (813.53–1469.69) | −0.14 (−0.17, −0.11) |
| Norway | 246 (185.69–330.67) | 252.58 (189.93–338.45) | 0.06 (0.05, 0.07) |
| Oman | 268.87 (198.22–364.89) | 276.45 (205.55–373.24) | 0.13 (−0.07, 0.32) |
| Pakistan | 864.24 (628.89–1191.17) | 875.34 (629.85–1227.23) | 0.45 (0.23, 0.67) |
| Palau | 921.05 (688.98–1266.77) | 964.74 (719.94–1311.16) | 0.14 (0.09, 0.20) |
| Palestine | 273.05 (203.42–370.79) | 275.91 (205.55–377.5) | 0.00 (−0.01, 0.02) |
| Panama | 483.89 (363.29–657.29) | 566.52 (465.97–684.08) | 0.85 (0.67, 1.03) |
| Papua New Guinea | 2013.82 (1535.05–2681.72) | 1854.81 (1376.26–2559.36) | −1.02 (−1.34, −0.71) |
| Paraguay | 795.54 (598.24–1088.75) | 987.64 (674.76–1432.07) | 1.38 (1.04, 1.73) |
| Peru | 698.93 (532.09–941.23) | 598.99 (461.34–784.58) | −0.90 (−1.08, −0.72) |
| Philippines | 390.3 (292.69–530.4) | 442.02 (332.1–600.69) | 0.67 (0.54, 0.80) |
| Poland | 146.21 (111–194.93) | 140.42 (106.08–188.93) | −0.17 (−0.23, −0.12) |
| Portugal | 213.61 (160.51–290.79) | 206.18 (152.94–281.14) | −0.18 (−0.22, −0.14) |
| Puerto Rico | 496.97 (376.58–664.94) | 492.97 (372.96–662.38) | −0.02 (−0.06, 0.02) |
| Qatar | 421.9 (311.15–572.87) | 441.72 (321.4–605.04) | 0.30 (0.20, 0.40) |
| Republic of Korea | 235.57 (183.8–307.49) | 268.46 (202.68–364.37) | 0.93 (0.74, 1.11) |
| Republic of Moldova | 195.97 (149.22–259.21) | 212.74 (176.22–257.98) | 0.63 (0.45, 0.82) |
| Romania | 229.64 (172.64–309.66) | 268.14 (199.83–343.63) | 0.76 (0.55, 0.97) |
| Russian Federation | 174.24 (132.88–230.7) | 168.04 (128.7–221.18) | −0.19 (−0.22, −0.15) |
| Rwanda | 1798.19 (1377.3–2360.01) | 1325.08 (1035.38–1747.44) | −1.27 (−1.39, −1.15) |
| Saint Kitts and Nevis | 538.23 (411.78–724.68) | 522.79 (399.03–705.93) | −0.13 (−0.16, −0.09) |
| Saint Lucia | 768.74 (578.17–1054.65) | 825.4 (649–1066.96) | 0.50 (0.35, 0.66) |
| Saint Vincent and the Grenadines | 594.76 (457.52–799.04) | 530.12 (402–714.48) | −0.45 (−0.57, −0.33) |
| Samoa | 718.02 (534.18–974.95) | 709.48 (529.83–961.33) | −0.05 (−0.08, −0.02) |
| San Marino | 211.75 (156.89–291.74) | 201.57 (150.21–275.04) | −0.20 (−0.23, −0.17) |
| Sao Tome and Principe | 1304.69 (982.55–1760.04) | 1247.15 (955.03–1624.61) | −0.27 (−0.39, −0.15) |
| Saudi Arabia | 261.04 (193.32–346.52) | 256.16 (189.9–348.67) | −0.10 (−0.13, −0.07) |
| Senegal | 1959.51 (1469.48–2608.32) | 1874.24 (1398.08–2515.61) | −0.38 (−0.46, −0.30) |
| Serbia | 126.55 (95.57–167.05) | 127.12 (96.88–169.83) | 0.00 (−0.02, 0.02) |
| Seychelles | 274.84 (205.9–370.58) | 273.33 (201.78–370.75) | −0.01 (−0.07, 0.06) |
| Sierra Leone | 1365.72 (1038.35–1828.3) | 1309.26 (986.57–1770.76) | −0.22 (−0.30, −0.14) |
| Singapore | 252.71 (192.59–340.02) | 249.45 (185.36–337.71) | −0.03 (−0.07, 0.02) |
| Slovakia | 110.07 (84.84–145.59) | 112.3 (84.97–149.2) | 0.06 (0.04, 0.08) |
| Slovenia | 125.53 (95.96–169.12) | 126.22 (95.2–170.94) | 0.04 (0.01, 0.07) |
| Solomon Islands | 1838.6 (1376.56–2463.92) | 1802.46 (1343.39–2446.27) | 0.03 (−0.06, 0.12) |
| Somalia | 2393.81 (1906.82–3047.6) | 2236.44 (1731.71–2905.22) | −0.15 (−0.30, 0.00) |
| South Africa | 3969.28 (3182.04–4999.74) | 2788.41 (2061.49–3784.93) | −1.36 (−2.06, −0.67) |
| South Sudan | 2657.13 (2053.26–3494.96) | 2486.48 (1899.77–3310.65) | 0.35 (0.20, 0.50) |
| Spain | 219.37 (164.31–296.87) | 208.58 (155.27–280.84) | −0.23 (−0.26, −0.20) |
| Sri Lanka | 83.98 (64.59–110.14) | 75.87 (57.14–101.04) | −0.77 (−1.20, −0.34) |
| Sudan | 645.23 (475.54–886.59) | 730.44 (530.73–1012.94) | −0.28 (−0.61, 0.05) |
| Suriname | 353.69 (272.58–469.16) | 347.31 (262.67–462.06) | −0.08 (−0.10, −0.07) |
| Sweden | 234.45 (172.97–314.72) | 233.07 (173.5–318.78) | −0.05 (−0.09, −0.02) |
| Switzerland | 211.9 (158.69–285.5) | 210.58 (157.21–287.28) | −0.03 (−0.07, 0.01) |
| Syrian Arab Republic | 282.5 (212.35–382.71) | 260.92 (196.78–350.71) | −0.27 (−0.33, −0.22) |
| Taiwan (Province of China) | 325.21 (248.57–438.97) | 320.95 (241.27–434.21) | −0.13 (−0.17, −0.08) |
| Tajikistan | 189.95 (146.55–248.63) | 164.29 (127.29–213.84) | −0.78 (−0.89, −0.67) |
| Thailand | 469.96 (340.78–638.3) | 439.25 (324.55–602.12) | −0.99 (−1.32, −0.65) |
| Timor−Leste | 344.85 (258.83–467.1) | 352.66 (266.9–471.18) | 0.09 (0.04, 0.14) |
| Togo | 1361.04 (1023.77–1830.84) | 1182.19 (897.08–1578.7) | −1.40 (−1.72, −1.07) |
| Tokelau | 1061.54 (790.41–1442.32) | 1061.71 (794.25–1457.12) | 0.01 (−0.02, 0.04) |
| Tonga | 1261.66 (947.75–1734.75) | 1263.97 (939.61–1757.27) | 0.00 (−0.04, 0.05) |
| Trinidad and Tobago | 409.67 (313.47–553.49) | 379.7 (299.5–484.42) | −0.39 (−0.49, −0.29) |
| Tunisia | 271.03 (201.92–369) | 268.03 (200.65–364.04) | −0.04 (−0.07, −0.02) |
| Turkey | 149.41 (112.32–198.36) | 148.14 (109.47–196.06) | −0.02 (−0.23, 0.19) |
| Turkmenistan | 179.11 (137.16–238.44) | 170.85 (128.67–230.15) | −0.25 (−0.31, −0.18) |
| Tuvalu | 897.87 (644.77–1279.34) | 925.69 (669.25–1307.27) | 0.01 (−0.02, 0.04) |
| Uganda | 2416.24 (1847.68–3257.14) | 3262.7 (2983.26–3617.31) | 0.31 (0.01, 0.60) |
| Ukraine | 220.44 (167.76–287.76) | 152.07 (115.29–203.65) | −2.07 (−2.42, −1.71) |
| United Arab Emirates | 292.52 (216.83–397.14) | 288.9 (212.83–390.56) | 0.04 (−0.01, 0.10) |
| United Kingdom | 246.97 (185.59–332.24) | 242.96 (181.28–329.66) | 0.07 (0.01, 0.13) |
| United Republic of Tanzania | 4316.68 (3915.74–4715.29) | 1984.41 (1620.45–2430.12) | −3.49 (−3.92, −3.06) |
| United States of America | 245.5 (187.55–323.96) | 255.27 (193.67–341.08) | 0.13 (−0.09, 0.35) |
| United States Virgin Islands | 528.95 (403.66–716.25) | 519.78 (391.96–704.14) | −0.04 (−0.06, −0.02) |
| Uruguay | 460.11 (344.56–641.17) | 409.34 (359.82–466.84) | 0.03 (−0.12, 0.19) |
| Uzbekistan | 183.47 (140.48–244) | 166.25 (128.39–223.11) | −0.57 (−0.66, −0.49) |
| Vanuatu | 1132.29 (849.52–1530.27) | 1108.45 (825–1519.77) | −0.07 (−0.14, −0.01) |
| Venezuela (Bolivarian Republic of) | 671.11 (493.88–933.3) | 848.21 (624.5–1057.47) | 1.09 (0.87, 1.30) |
| Viet Nam | 249.22 (185–339.86) | 256.96 (190.58–351.56) | 0.07 (0.03, 0.11) |
| Yemen | 714.5 (530.44–987.81) | 729.04 (535.4–1004.31) | −0.33 (−0.51, −0.16) |
| Zambia | 3519.39 (2918.71–4311.88) | 2906.46 (2357.6–3569.33) | −1.49 (−1.90, −1.09) |
| Zimbabwe | 1500.37 (1135.08–2015.25) | 1384.62 (1122.36–1741.54) | −0.28 (−0.34, −0.22) |

**Abbreviations:** ASR, age-standardized rate; UI, uncertainty intervals; CI, confidence interval.
