# Supplementary material for: Evaluating the global, regional, and national impact of syphilis: results from the global burden of disease study 2019
Source: Sci Rep. 2023 Jul 14;13:11386. doi: 10.1038/s41598-023-38294-4 (PMC10349077; doi:10.1038/s41598-023-38294-4)
Supplement: Supplementary file 2 — Supplementary Table 2. [file 41598_2023_38294_MOESM2_ESM.doc]

Supplementary Table 2 DALYs of syphilis in 204 countries and territories in 1990 and 2019 and EAPC from 1990 to 2019

| 204 countries or territories | ASR of DALYs (1990) | ASR of DALYs (2019) | EAPC in ASR (1990–2019, 95% CI) |
| --- | --- | --- | --- |
| Afghanistan | 45.75 (13.33–106.55) | 47.33 (14.49–108.48) | −0.28 (−0.72, 0.16) |
| Albania | 22.35 (6.61–50.61) | 12.8 (3.77–28.5) | −2.18 (−2.42, −1.93) |
| Algeria | 39.98 (12.24–92.2) | 17.39 (5.53–37.8) | −3.42 (−4.27, −2.57) |
| American Samoa | 288.15 (93.94–580.01) | 208.22 (72.23–408.99) | −1.16 (−1.26, −1.05) |
| Andorra | 12.07 (3.64–27.63) | 9.06 (2.85–20.44) | −0.68 (−0.98, −0.38) |
| Angola | 534.8 (178.62–1165.13) | 397.21 (131.75–850.47) | −0.93 (−1.13, −0.74) |
| Antigua and Barbuda | 6.59 (5.18–8.41) | 2.13 (1.57–2.88) | −2.78 (−3.41, −2.15) |
| Argentina | 14.93 (12.57–17.52) | 4.99 (3.63–6.75) | −3.54 (−4.10, −2.98) |
| Armenia | 5.77 (1.3–7.04) | 0.12 (0.07–0.49) | −18.92 (−21.40, −16.44) |
| Australia | 1.08 (0.89–1.31) | 0.26 (0.21–0.35) | −3.81 (−4.71, −2.91) |
| Austria | 0.97 (0.75–1.19) | 0.48 (0.35–0.64) | −1.39 (−1.87, −0.91) |
| Azerbaijan | 32.08 (12.22–66.26) | 22.68 (6.84–51.29) | −1.66 (−2.00, −1.32) |
| Bahamas | 2.26 (1.83–2.79) | 1.25 (0.97–1.64) | −1.55 (−1.91, −1.19) |
| Bahrain | 20.06 (5.83–46.97) | 19.24 (6.05–42.34) | −0.06 (−0.52, 0.40) |
| Bangladesh | 254.16 (81.12–544.1) | 129.84 (41.1–286.44) | −3.19 (−3.50, −2.89) |
| Barbados | 8.23 (6.45–10.37) | 2.6 (1.89–3.54) | −3.27 (−3.85, −2.70) |
| Belarus | 3.39 (2.19–4.37) | 0.38 (0.25–0.64) | −10.06 (−10.94, −9.19) |
| Belgium | 0.83 (0.59–1.04) | 0.31 (0.22–0.42) | −3.09 (−3.58, −2.60) |
| Belize | 2.31 (1.8–2.91) | 0.81 (0.65–1.01) | −3.19 (−3.70, −2.68) |
| Benin | 143.33 (46.9–307) | 78.22 (26.19–165.48) | −3.80 (−4.42, −3.18) |
| Bermuda | 1.7 (1.43–2.03) | 0.59 (0.46–0.79) | −2.78 (−3.44, −2.13) |
| Bhutan | 297.46 (91.32–667.86) | 278.64 (79.81–643.45) | −0.86 (−1.21, −0.51) |
| Bolivia (Plurinational State of) | 295.57 (89.15–659.11) | 122.36 (42.87–245.94) | −4.33 (−4.79, −3.88) |
| Bosnia and Herzegovina | 17.15 (5.08–38.63) | 9.69 (2.76–22.01) | −1.95 (−2.65, −1.25) |
| Botswana | 637.83 (249.13–1249.15) | 287.76 (93.21–635.78) | −4.48 (−5.95, −3.01) |
| Brazil | 25.94 (21.08–32.51) | 12.85 (10.27–15.81) | −1.81 (−2.66, −0.96) |
| Brunei Darussalam | 16.03 (6.38–32.01) | 14.13 (4.63–31.14) | −0.11 (−0.32, 0.10) |
| Bulgaria | 0.37 (0.31–0.44) | 0.18 (0.14–0.23) | −3.45 (−5.09, −1.82) |
| Burkina Faso | 192.33 (63.22–404.64) | 148.89 (52.71–310.51) | −0.24 (−0.58, 0.10) |
| Burundi | 81.19 (34.78–152.83) | 39.49 (17.78–74.37) | −3.00 (−3.44, −2.56) |
| Cabo Verde | 123.26 (39.73–273.35) | 52.92 (16.71–119.62) | −4.10 (−4.65, −3.55) |
| Cambodia | 19.5 (6.61–43.38) | 7.99 (2.75–17.74) | −5.79 (−6.83, −4.75) |
| Cameroon | 657.94 (222.49–1368.31) | 277.61 (87.08–612.77) | −2.50 (−3.04, −1.96) |
| Canada | 1.01 (0.77–1.26) | 0.63 (0.46–0.85) | −1.24 (−1.53, −0.96) |
| Central African Republic | 550.75 (190.89–1158.59) | 447.53 (155.93–924.48) | −1.77 (−2.07, −1.46) |
| Chad | 481.01 (158.57–1027.48) | 300.76 (98.38–625.26) | −2.25 (−2.69, −1.82) |
| Chile | 3.76 (3.19–4.32) | 0.52 (0.4–0.81) | −5.28 (−6.10, −4.46) |
| China | 34.37 (11.38–72.92) | 22.51 (7.03–51.27) | −0.77 (−1.11, −0.43) |
| Colombia | 16.02 (13.42–19) | 4.65 (3.2–6.62) | −3.95 (−4.25, −3.66) |
| Comoros | 429.3 (140.35–959.57) | 186.4 (60.72–416.88) | −3.75 (−4.09, −3.42) |
| Congo | 361.34 (122.05–751.5) | 229.3 (77.62–481.02) | −1.86 (−2.15, −1.57) |
| Cook Islands | 194.08 (59.76–426) | 114.9 (49.41–190.18) | −1.73 (−2.01, −1.45) |
| Costa Rica | 5.58 (4.72–6.6) | 2.91 (2.1–3.92) | −1.61 (−2.59, −0.63) |
| Côte d'Ivoire | 368.19 (127.03–768.79) | 199.81 (65–439.43) | −2.06 (−2.37, −1.74) |
| Croatia | 0.26 (0.21–0.31) | 0.12 (0.09–0.17) | −2.38 (−2.87, −1.89) |
| Cuba | 0.56 (0.45–0.72) | 0.94 (0.73–1.21) | 2.21 (1.11, 3.31) |
| Cyprus | 11.23 (3–29.67) | 4.85 (1.53–11.09) | −3.12 (−3.49, −2.76) |
| Czechia | 0.59 (0.28–0.71) | 0.13 (0.1–0.18) | −4.13 (−5.16, −3.10) |
| Democratic People's Republic of Korea | 28.94 (8.42–73.18) | 24.03 (7.18–52.52) | −0.55 (−0.83, −0.26) |
| Democratic Republic of the Congo | 687.95 (239.1–1358.61) | 345.01 (122.86–726.97) | −3.28 (−3.68, −2.87) |
| Denmark | 0.24 (0.19–0.32) | 0.15 (0.11–0.22) | −1.60 (−2.10, −1.11) |
| Djibouti | 215.72 (79.13–429.19) | 177.96 (65–372.12) | −0.93 (−1.26, −0.61) |
| Dominica | 145.78 (49.47–314.58) | 147.97 (43.4–337.37) | 0.29 (−0.03, 0.60) |
| Dominican Republic | 142.45 (48.99–303.58) | 152.94 (52.49–323.06) | −0.50 (−0.92, −0.07) |
| Ecuador | 152.1 (46.2–340.52) | 88.95 (25.43–203.69) | −1.99 (−2.12, −1.85) |
| Egypt | 18.26 (5.52–41.74) | 10.61 (2.75–26.09) | −2.25 (−2.45, −2.05) |
| El Salvador | 29.78 (9.52–67.66) | 14.42 (4.59–32.87) | −3.17 (−3.51, −2.84) |
| Equatorial Guinea | 747.57 (246.71–1607.39) | 727.02 (258.1–1474.34) | 0.00 (−0.13, 0.12) |
| Eritrea | 206.96 (78.12–423.57) | 102.61 (36.5–213.17) | −2.10 (−2.53, −1.68) |
| Estonia | 5.85 (0.39–7.6) | 0.12 (0.08–0.19) | −10.11 (−13.60, −6.61) |
| Eswatini | 714.94 (259.7–1445.68) | 241.02 (81.04–510.6) | −3.36 (−3.73, −2.98) |
| Ethiopia | 849.19 (289.56–1781.9) | 223.33 (78.59–478.9) | −5.13 (−5.44, −4.82) |
| Fiji | 127.02 (41.99–265.51) | 124.18 (36.86–275.23) | −0.13 (−0.33, 0.07) |
| Finland | 0.41 (0.27–0.5) | 0.2 (0.14–0.28) | −1.81 (−2.20, −1.42) |
| France | 0.65 (0.54–0.76) | 0.23 (0.17–0.31) | −3.10 (−3.57, −2.62) |
| Gabon | 368.56 (129.01–818.23) | 183.07 (59.94–401.05) | −3.82 (−4.42, −3.22) |
| Gambia | 336.86 (105.63–746.64) | 223.5 (72.88–495.32) | −2.41 (−2.84, −1.98) |
| Georgia | 1.11 (0.48–1.38) | 0.27 (0.21–0.37) | −1.07 (−3.25, 1.11) |
| Germany | 0.76 (0.57–0.89) | 0.25 (0.19–0.34) | −3.61 (−4.18, −3.03) |
| Ghana | 173.04 (54.83–401.82) | 162.81 (53.61–359.64) | −0.73 (−1.28, −0.17) |
| Greece | 0.33 (0.26–0.4) | 0.18 (0.13–0.26) | −1.44 (−1.81, −1.06) |
| Greenland | 12.22 (4.22–26.17) | 9.12 (3.08–21.07) | −0.92 (−1.05, −0.79) |
| Grenada | 7.58 (6.03–9.37) | 1.83 (1.46–2.28) | −3.51 (−4.02, −2.99) |
| Guam | 221.38 (73.78–443.58) | 209.87 (66.65–433.07) | −0.18 (−0.24, −0.12) |
| Guatemala | 5.87 (4.85–6.83) | 1.32 (1.05–1.65) | −4.77 (−5.15, −4.39) |
| Guinea | 367.49 (119.43–781.48) | 213.61 (65.49–458.08) | −2.54 (−3.14, −1.94) |
| Guinea−Bissau | 913.75 (281.91–1972.04) | 374.78 (119.14–812.07) | −3.88 (−5.14, −2.61) |
| Guyana | 23.45 (17.32–32.07) | 5.21 (3.5–7.56) | −3.86 (−4.45, −3.27) |
| Haiti | 455.05 (159.77–978.32) | 416.52 (154.29–846) | −0.53 (−0.98, −0.07) |
| Honduras | 36.01 (11.82–79.14) | 18.63 (6.13–41.74) | −3.67 (−4.19, −3.15) |
| Hungary | 0.46 (0.25–0.55) | 0.12 (0.09–0.17) | −3.15 (−4.33, −1.98) |
| Iceland | 0.77 (0.51–0.92) | 0.19 (0.14–0.3) | −4.57 (−5.09, −4.04) |
| India | 113.51 (36.7–250.16) | 54.82 (17.38–119.46) | −3.57 (−4.57, −2.58) |
| Indonesia | 143.03 (43.93–325.37) | 172.26 (52.91–380.11) | 1.01 (0.64, 1.38) |
| Iran (Islamic Republic of) | 12.74 (4.01–30.39) | 8.72 (2.9–18.71) | −1.37 (−1.66, −1.08) |
| Iraq | 27.48 (8.44–61.43) | 21.44 (6.6–47.16) | −0.71 (−0.90, −0.52) |
| Ireland | 0.43 (0.32–0.53) | 0.17 (0.12–0.25) | −2.68 (−3.05, −2.31) |
| Israel | 0.47 (0.35–0.57) | 0.19 (0.14–0.27) | −3.78 (−4.55, −3.02) |
| Italy | 1.57 (1.39–1.77) | 0.48 (0.37–0.62) | −4.77 (−5.28, −4.26) |
| Jamaica | 3.18 (2.54–3.96) | 1.74 (1.26–2.35) | −0.54 (−1.66, 0.58) |
| Japan | 0.76 (0.63–0.91) | 0.39 (0.3–0.52) | −2.12 (−2.41, −1.82) |
| Jordan | 37.45 (11.3–87.54) | 29.63 (8.89–68.46) | −0.96 (−1.18, −0.73) |
| Kazakhstan | 2.58 (2.12–3.05) | 0.63 (0.47–0.89) | −6.83 (−8.89, −4.77) |
| Kenya | 298.01 (100.5–635.1) | 129.81 (44.3–277.06) | −2.94 (−3.29, −2.59) |
| Kiribati | 305.33 (98.22–647.13) | 306.09 (96.07–640.74) | 0.32 (0.15, 0.49) |
| Kuwait | 0.24 (0.18–0.35) | 0.14 (0.09–0.26) | −2.39 (−3.16, −1.61) |
| Kyrgyzstan | 13.81 (9.54–21.58) | 0.47 (0.35–0.68) | −13.95 (−16.67, −11.24) |
| Lao People's Democratic Republic | 49.67 (15.37–109.37) | 35.83 (10.56–83.26) | −1.20 (−1.42, −0.98) |
| Latvia | 3.48 (1.45–4.21) | 0.17 (0.12–0.31) | −16.04 (−18.72, −13.36) |
| Lebanon | 14.26 (4.37–32.15) | 13.11 (3.63–30.66) | −1.01 (−1.32, −0.70) |
| Lesotho | 464.69 (161.3–937.81) | 268.55 (87.06–600.38) | −1.31 (−1.59, −1.04) |
| Liberia | 820.19 (263.53–1768.06) | 717.24 (258.12–1415.6) | 0.33 (−0.27, 0.94) |
| Libya | 17.26 (5.36–38.93) | 16.1 (5.11–35.63) | 0.46 (0.10, 0.82) |
| Lithuania | 2.07 (0.65–2.51) | 0.12 (0.09–0.19) | −10.33 (−12.43, −8.24) |
| Luxembourg | 2.66 (2.03–3.14) | 0.51 (0.4–0.73) | −5.47 (−6.26, −4.68) |
| Madagascar | 689.17 (245.07–1388.82) | 558.61 (208.2–1082.55) | −1.15 (−1.64, −0.65) |
| Malawi | 453.73 (167.82–911.32) | 451.95 (166.05–919.71) | −0.76 (−1.64, 0.13) |
| Malaysia | 12.46 (4.14–27.14) | 6.13 (2.17–13.59) | −2.99 (−3.33, −2.65) |
| Maldives | 27.86 (9.3–60.77) | 13.61 (4.38–30.04) | −2.24 (−2.55, −1.93) |
| Mali | 499.75 (152.73–1120.9) | 408.51 (141.99–822.61) | −0.03 (−0.41, 0.34) |
| Malta | 0.31 (0.25–0.4) | 0.13 (0.09–0.21) | −2.79 (−3.23, −2.35) |
| Marshall Islands | 242.32 (77.3–496.88) | 235.61 (78.18–476.11) | 0.15 (−0.13, 0.43) |
| Mauritania | 455.56 (151.33–964.02) | 260.01 (84.32–557.79) | −1.56 (−1.81, −1.31) |
| Mauritius | 0.7 (0.49–0.85) | 0.27 (0.19–0.43) | −1.00 (−2.26, 0.25) |
| Mexico | 5.73 (5.09–6.37) | 1.31 (1.12–1.53) | −4.33 (−5.27, −3.39) |
| Micronesia (Federated States of) | 339.62 (112.04–732.92) | 246.24 (89.71–476.63) | −0.90 (−1.10, −0.70) |
| Monaco | 11.12 (3.56–25.83) | 7.1 (2.38–15.86) | −1.73 (−1.87, −1.58) |
| Mongolia | 121.73 (33–272.18) | 183.81 (48.45–391.89) | 2.36 (1.73, 2.98) |
| Montenegro | 14.52 (4.5–33.39) | 11.72 (3.73–25.93) | −0.89 (−1.03, −0.76) |
| Morocco | 101.7 (31.48–230.26) | 64.84 (18.16–150.6) | −4.01 (−5.20, −2.82) |
| Mozambique | 1408.78 (500.27–2864.53) | 418.46 (150.26–864.12) | −4.09 (−4.43, −3.76) |
| Myanmar | 72.95 (21.87–170.29) | 89.1 (28.72–195.65) | 0.70 (0.47, 0.93) |
| Namibia | 325.17 (106.75–681.78) | 209.72 (70.38–438.97) | −3.03 (−4.02, −2.05) |
| Nauru | 181.01 (56.51–411.33) | 226.96 (69.25–505.27) | 0.97 (0.67, 1.27) |
| Nepal | 160.05 (49.2–358) | 124.1 (37.64–266.4) | −1.02 (−1.11, −0.94) |
| Netherlands | 0.76 (0.44–0.89) | 0.23 (0.18–0.32) | −4.35 (−5.07, −3.63) |
| New Zealand | 0.46 (0.33–0.57) | 0.27 (0.2–0.37) | −1.07 (−1.66, −0.48) |
| Nicaragua | 40.1 (12.77–86.36) | 13.75 (4.69–29.34) | −5.14 (−6.32, −3.96) |
| Niger | 74.26 (26.73–161.12) | 31.66 (11.16–67.19) | −4.06 (−4.65, −3.46) |
| Nigeria | 196.6 (61.18–430.33) | 218.12 (70.01–497.51) | 0.94 (0.58, 1.31) |
| Niue | 202.13 (63.29–441.2) | 226.93 (73.47–498.64) | 0.58 (0.47, 0.68) |
| North Macedonia | 13.48 (3.94–30.76) | 11.96 (3.56–27.21) | −0.23 (−0.44, −0.01) |
| Northern Mariana Islands | 179.61 (59.64–377.61) | 178.88 (60.5–354.81) | 0.45 (0.20, 0.71) |
| Norway | 0.81 (0.6–1.2) | 1.2 (0.79–1.48) | 1.79 (1.54, 2.04) |
| Oman | 8.9 (2.81–20.44) | 4.77 (1.61–10.61) | −3.27 (−3.70, −2.84) |
| Pakistan | 186.14 (54.59–421.63) | 131.88 (41.73–290.19) | −0.28 (−0.71, 0.15) |
| Palau | 214.89 (68.64–466.37) | 263.83 (94.05–516.65) | 1.08 (0.92, 1.24) |
| Palestine | 19.08 (5.95–42.44) | 16.09 (4.97–34.54) | −0.40 (−0.55, −0.25) |
| Panama | 1.53 (1.23–1.87) | 2.31 (1.71–3.06) | 3.51 (2.46, 4.56) |
| Papua New Guinea | 595.93 (200.64–1276.64) | 499.49 (159.63–1042.53) | −1.31 (−1.60, −1.02) |
| Paraguay | 289.74 (93.59–606.1) | 320.53 (113.78–641.59) | 0.49 (0.21, 0.77) |
| Peru | 153.37 (46.57–328.76) | 54.13 (16.53–124.3) | −5.30 (−5.77, −4.83) |
| Philippines | 70.19 (21.56–153.76) | 78.14 (23.29–174.32) | 0.28 (0.06, 0.49) |
| Poland | 1.15 (0.77–1.33) | 0.39 (0.29–0.5) | −3.51 (−3.99, −3.03) |
| Portugal | 2.53 (2.07–3) | 0.31 (0.22–0.41) | −7.53 (−9.70, −5.36) |
| Puerto Rico | 1.09 (0.93–1.4) | 0.76 (0.56–1) | −1.49 (−2.34, −0.64) |
| Qatar | 23.31 (6.74–53.04) | 16.78 (4.99–37.18) | −1.56 (−1.76, −1.36) |
| Republic of Korea | 16.75 (5.29–37.81) | 18.61 (6.15–40.05) | 1.10 (0.82, 1.37) |
| Republic of Moldova | 2.04 (1.58–2.46) | 0.26 (0.2–0.39) | −7.97 (−8.99, −6.94) |
| Romania | 0.87 (0.74–1.02) | 0.36 (0.28–0.48) | −5.64 (−8.24, −3.04) |
| Russian Federation | 2.45 (2.21–2.73) | 1.05 (0.88–1.24) | −5.13 (−6.80, −3.46) |
| Rwanda | 243.34 (88.84–506.2) | 149.52 (55.72–317.01) | −1.56 (−1.96, −1.17) |
| Saint Kitts and Nevis | 16.2 (13.41–19.75) | 3.59 (2.61–4.74) | −3.89 (−4.79, −3.00) |
| Saint Lucia | 18.24 (14.66–22.46) | 5.17 (3.65–7.18) | −3.42 (−4.04, −2.80) |
| Saint Vincent and the Grenadines | 47.12 (33.49–62.16) | 6.96 (5.2–9.26) | −4.19 (−5.37, −3.01) |
| Samoa | 257.9 (81.25–549.84) | 240.52 (78.88–515.5) | −0.18 (−0.54, 0.18) |
| San Marino | 8.28 (2.49–20.47) | 6.69 (2.19–14.74) | −0.63 (−0.87, −0.38) |
| Sao Tome and Principe | 193.12 (61.08–421.4) | 123.63 (38.52–273.48) | −1.99 (−2.67, −1.32) |
| Saudi Arabia | 12.61 (3.95–29.32) | 5.55 (1.77–11.81) | −2.85 (−3.05, −2.66) |
| Senegal | 131.82 (45.34–276.61) | 68.19 (22.91–143.86) | −3.89 (−4.52, −3.26) |
| Serbia | 16.19 (4.71–36.65) | 9.33 (2.85–20.83) | −1.90 (−2.11, −1.69) |
| Seychelles | 17.04 (6.49–34.81) | 14.53 (5.24–31.83) | −0.33 (−0.46, −0.21) |
| Sierra Leone | 291.11 (88.21–631.98) | 174.37 (52.53–394.02) | −2.01 (−2.28, −1.75) |
| Singapore | 3.41 (1.14–4.15) | 0.39 (0.28–0.52) | −6.46 (−7.96, −4.96) |
| Slovakia | 11.31 (3.47–25.3) | 9.1 (2.68–20.48) | −0.52 (−0.68, −0.37) |
| Slovenia | 0.18 (0.13–0.23) | 0.08 (0.06–0.13) | −2.41 (−2.74, −2.07) |
| Solomon Islands | 836.79 (296.8–1645.2) | 920.93 (384.81–1597.21) | 0.97 (0.61, 1.33) |
| Somalia | 354.63 (132.05–724.3) | 284.58 (104.67–571.49) | 0.04 (−0.43, 0.52) |
| South Africa | 683 (244.34–1376.4) | 404.41 (136.24–822.68) | −1.79 (−2.32, −1.25) |
| South Sudan | 658.08 (229.06–1416.7) | 564.29 (176.91–1247.54) | 0.69 (0.35, 1.03) |
| Spain | 2.54 (1.86–2.94) | 0.32 (0.25–0.42) | −7.08 (−8.02, −6.15) |
| Sri Lanka | 11.36 (3.87–24.29) | 6.13 (2.02–13.97) | −2.64 (−3.11, −2.16) |
| Sudan | 159.25 (44.01–382.45) | 157.9 (44.49–379.81) | −1.04 (−1.57, −0.50) |
| Suriname | 196.29 (59.52–435.51) | 126.36 (36.3–285.08) | −1.75 (−1.89, −1.61) |
| Sweden | 0.34 (0.19–0.44) | 0.16 (0.11–0.24) | −2.10 (−2.53, −1.67) |
| Switzerland | 0.49 (0.37–0.64) | 0.35 (0.27–0.45) | −1.19 (−1.50, −0.88) |
| Syrian Arab Republic | 27.52 (8.08–68.7) | 16.06 (5.12–34.76) | −1.01 (−1.33, −0.69) |
| Taiwan (Province of China) | 1.14 (0.94–1.39) | 0.98 (0.75–1.26) | −0.50 (−1.02, 0.02) |
| Tajikistan | 23.57 (11.04–44.08) | 14.14 (6.69–26.88) | −2.90 (−3.45, −2.35) |
| Thailand | 25.94 (8.45–57.43) | 7.17 (2.62–15.25) | −7.17 (−8.63, −5.72) |
| Timor−Leste | 79.93 (24.88–179.45) | 59.11 (19.3–131.8) | −1.15 (−1.30, −1.00) |
| Togo | 190.89 (61.26–430.54) | 84.17 (27.19–186.55) | −5.44 (−6.40, −4.49) |
| Tokelau | 191.9 (61.62–399.72) | 139.05 (47.58–274.47) | −0.83 (−1.06, −0.60) |
| Tonga | 199.44 (63.27–436.64) | 159.65 (50.53–335.12) | −0.89 (−1.07, −0.72) |
| Trinidad and Tobago | 0.58 (0.47–0.74) | 0.6 (0.46–0.79) | 0.24 (−0.68, 1.16) |
| Tunisia | 27.02 (7.76–61.29) | 13.3 (4.27–29.17) | −2.30 (−2.51, −2.10) |
| Turkey | 21.19 (6.23–48.63) | 11 (3.36–25.14) | −2.26 (−2.37, −2.14) |
| Turkmenistan | 1.84 (0.74–2.2) | 0.21 (0.15–0.34) | −9.58 (−11.48, −7.68) |
| Tuvalu | 319.05 (95.21–752.51) | 217.66 (76.44–431.45) | −1.36 (−1.52, −1.20) |
| Uganda | 388.7 (129.35–840.76) | 712.32 (273.83–1383.54) | 1.30 (0.68, 1.92) |
| Ukraine | 4.88 (1.48–5.73) | 0.44 (0.29–1.37) | −16.32 (−19.86, −12.78) |
| United Arab Emirates | 10.81 (3.44–25.04) | 4.72 (1.78–9.93) | −4.18 (−4.85, −3.51) |
| United Kingdom | 11.76 (3.65–26.59) | 11.07 (3.36–23.79) | 1.09 (0.65, 1.53) |
| United Republic of Tanzania | 824.81 (309.93–1622.92) | 348.27 (126.73–696.9) | −3.42 (−3.84, −3.01) |
| United States of America | 1.82 (1.52–2.21) | 1.03 (0.82–1.28) | −2.28 (−2.55, −2.02) |
| United States Virgin Islands | 109.01 (36.29–232.22) | 73.79 (24.19–164.38) | −1.37 (−1.43, −1.30) |
| Uruguay | 26.3 (19.52–34.75) | 0.97 (0.75–1.29) | −12.44 (−14.64, −10.25) |
| Uzbekistan | 3.45 (1.04–4.12) | 0.18 (0.11–0.48) | −14.96 (−17.17, −12.75) |
| Vanuatu | 323.96 (108.88–668.7) | 414.9 (147.3–801) | 0.41 (0.28, 0.54) |
| Venezuela (Bolivarian Republic of) | 6.08 (4.99–7.4) | 4.89 (3.32–7) | −1.73 (−2.53, −0.92) |
| Viet Nam | 44.82 (13.27–103.94) | 30.82 (9.52–66.76) | −1.88 (−2.05, −1.72) |
| Yemen | 81.47 (25.11–199.59) | 79.52 (24.6–180.08) | 0.04 (−0.14, 0.22) |
| Zambia | 559.97 (202.15–1103.83) | 410.5 (149.61–832.84) | −1.53 (−1.80, −1.25) |
| Zimbabwe | 202.08 (66.29–422.55) | 178.65 (63–367.56) | −0.25 (−0.42, −0.08) |

**Abbreviations:** DALYs, disability‐adjusted life years; ASR, age-standardized rate; UI, uncertainty intervals; CI, confidence interval.
